# Pan-cancer analysis of AIM2 inflammasomes with potential implications for immunotherapy in human cancer: A bulk omics research and single cell sequencing validation

**DOI:** 10.3389/fimmu.2022.998266

**Published:** 2022-09-29

**Authors:** Yan Qin, Liuxian Pan, Tianyu Qin, Hanyi Ruan, Yujie Zhang, Yan Zhang, Jianli Li, Jianrong Yang, Wei Li

**Affiliations:** ^1^ Department of Health Management, The People’s Hospital of Guangxi Zhuang Autonomous Region & Research center of Health Management, Guangxi Academy of Medical Sciences, Nanning, China; ^2^ Department of Medical Oncology, Guangxi Medical University Cancer Hospital, Nanning, China

**Keywords:** AIM2 inflammasomes, single-cell transcriptome sequencing, pan-cancer, immunotherapy, tumor microenvironment

## Abstract

**Background:**

The absent in melanoma 2 (AIM2) inflammasome is a multi-protein platform that recognizes aberrant cytoplasmic double-stranded DNA(dsDNA) and induces cytokine maturation, release, and pyroptosis. Some studies found that the AIM2 inflammasome was a double-edged sword in many cancers. However, there have been fewer studies on AIM2 inflammasomes in pan-cancer.

**Methods:**

Gene expression was analyzed using The Cancer Genome Atlas (TCGA) database and The Genotype-Tissue Expression (GTEx) database. Immunohistochemistry (IHC) was used to validate the expression of the AIM2. We used the survival curve to explore the prognostic significance of the AIM2 inflammasomes in pan-cancer. Mutations and methylation of AIM2 inflammasome-related genes (AIM2i-RGs) were also comprehensively analyzed. Single sample gene set enrichment analysis was used to calculate the AIM2 inflammasomes score and explore the correlation of the AIM2 inflammasomes score with immune-related genes and immune infiltrations. The function of AIM2 inflammasomes in pan-cancer was analyzed at the single-cell level. Single-cell transcriptome sequencing (scRNA-seq) data was used to assess the activation state of the AIM2 inflammasomes in the tumor microenvironment.

**Results:**

We found that AIM2i-RGs were aberrantly expressed in tumors and were strongly associated with prognosis. In pan-cancer, the expression of AIM2i-RGs was positively associated with copy number variation and negatively associated with methylation. In AIM2i-RGs, missense mutations were the predominant type of single nucleotide polymorphism. Moreover, we found that the drugs dimethyloxallyl glycine (DMOG) and Z-LNle-CHO may be sensitive to the AIM2 inflammasomes. The AIM2 inflammasomes score was significantly and positively correlated with the tumor immunity score and the stroma score. In most tumors, the AIM2 inflammasomes score was significantly and positively correlated with CD8+ T cell abundance in the tumor microenvironment. Additionally, the AIM2 inflammasomes score was significantly correlated with immune checkpoint genes in pan-cancer as well as immune checkpoint therapy-related markers including tumor mutational burden (TMB), microsatellite instability(MSI), and tumor immune dysfunction and exclusion(TIDE). scRNA-seq analysis suggested that AIM2 inflammasomes differ significantly among different cells in the tumor microenvironment. IHC confirmed low expression of AIM2 in colorectal cancer.

**Discussion:**

AIM2 inflammasomes may be a new target for future tumor therapy It is likely involved in tumor development, and its high expression may serve as a predictor of tumor immunotherapy efficacy.

## Introduction

Tumors, one of the causes of high morbidity and mortality, accounted for 740,000 deaths worldwide in 2021, that is, approximately >13% of all deaths (1). In the course of cancer research, the relationship between cancer and immune cells has gradually gained attention as the seventh major feature, in addition to the six major features such as self-sufficiency of cancer growth signals, unlimited replication potential, evasion of apoptosis, insensitivity to anti-growth signals, sustained angiogenesis, and tissue invasion/metastasis. In recent years, the emergence of cancer immunotherapy has transformed cancer treatment by modulating the relationship between the human immune system and cancer (2).Unfortunately, most people show limited or no response to it, and the effectiveness of cancer immunotherapy still needs to be improved (3).Therefore, it is valuable to identify people who are sensitive to immunotherapy prior to treatment. Therefore, there is an urgent need to find a molecular biomarker that can predict clinical outcomes and rate the effectiveness of immunotherapy.

In the study of the relationship between cancer and inflammation, it was found that inflammation plays a critical role in the occurrence, development, progression, angiogenesis, and invasion of cancer (4). Inflammasomes are multimeric protein complexes that form in cells (5) and play an important role in host defense (6), auto-inflammation (7), cancer (8), neurodegenerative diseases, cardiovascular diseases, obesity and associated metabolic syndrome (9). After recognizing the double-stranded DNA (ds DNA), absent in melanoma 2 (AIM2) inflammasomes, the prototype, and most characteristic member of the AIM2-like receptor (ALR) induce the activation of cysteine protease caspase-1 by aggregating an articulated protein ASC (CARD-containing apoptosis-associated specific protein), and this activation can lead to the inflammatory cytokine interleukin (IL)-1β and pre-IL-18 maturation and release, which eventually becomes active (5). Early studies found that the overexpression of AIM2 could reverse the melanoma phenotype (10).Therefore, AIM2 has always been present as a tumor suppressor. In contrast, AIM2 is thought to be an oncogenic factor in squamous cell carcinoma (SCC) of the skin and endometrial cancers (11, 12). In the absence of AIM2, the growth of SCC is inhibited by inducing cell cycle regulatory genes that inhibit cell death and promote cell proliferation (12). The mechanism may be due to the indirect effect of cytokines released by SCC cells on the activation of AIM2 inflammasomes. Because of the different immune responses of AIM2 inflammasomes, they are considered a double-edged sword in many cancers. For example, in AIM2 overexpression and silencing in hepatocellular carcinoma cells (HCC) studies, the anticancer effect of AIM2 was mediated by inflammasomes and induced by pyroptosis. Hepatitis B virus x protein-induced AIM2 deficiency was associated with increased cell migration and invasion (13, 14).In contrast, AIM2 gene deletion resulted in reduced liver injury and tumor development in a diethylnitrosamine-induced mouse model of hepatocellular carcinoma, suggesting that AIM2 plays a deleterious role in the development of HCC (15). Finally, AIM2 inflammasome is low expressed in Melanoma (16, 17), Colon cancer (18), Hepatocellular carcinoma (14, 15), Renal carcinoma (19, 20), Breast cancer (21), Prostate cancer (22, 23), playing a tumor suppressive role. It is highly expressed in SCC and plays a tumor promoting role. What’s more, AIM2 inflammasome inhibits the proliferation of cancer cells in glioblastoma multiform (16).

Although the role of AIM2 have been reported in limited types of cancers. However, there have not been many studies on AIM2 in pan-cancer yet. We performed a pan-cancer analysis of AIM2 in multiple cancer types using The Cancer Genome Atlas (TCGA) database. We further explored the association of AIM2 in cancer through prognosis analysis, immune infiltration analysis, methylation level analysis, and tumor purity. These findings provide important insights for future studies on AIM2 in pan-cancer.

## Method

### Single-cell transcriptome sequencing data analysis

The preparation and data analysis of scRNA-Seq was performed as previously described (24). The single-cell sequencing datasets was downloaded from GEO database (GSE152938). In brief, fresh tumor samples were obtained from the operating room to the laboratory in cold Hank’s balanced salt solution. After the samples were washed and were cut into 2–4 mm pieces. The tissue species were digested for 30 min at 37°C with gentle agitation in a digestion solution in HBSS. Samples were washed, filtered, erythrocytes removed and cell viability assayed before single cell sequencing. Two kidney renal clear cell carcinoma (KIRC) samples were collected from patients undergoing radical nephrectomy. The patients were not receiving any anti-tumor treatment therapy prior to sampling, including chemotherapy, radiotherapy, immunotherapy and Chinese medicine. All samples were sequenced using the Hiseq X10 (Illumina, San Diego, CA) with standard parameters. Preliminary sequencing files (.bcl) were converted to FASTQ files on CellRanger (version 3.0.2). R (version 3.5.2) and Seurat R package (version 3.1.1) were used for Quality Control (QC) and secondary analysis.

### Paraffin-embedded tissue collection

Paired cancers and paracancerous tissues were derived from 114 colon cancer patients from the Affiliated Cancer Hospital of Guangxi Medical University. There were 64 males and 50 females with a mean age of 65.57 years, stage I-II 46 cases and stage III-IV 68 cases. All patients were diagnosed with colon cancer and had not received chemotherapy or radiotherapy before tissue collection. Tumor specimens are all derived from surgically excised specimens and the time between isolation and fixation does not exceed 30 minutes. Written informed consent was acquired from all patients. The study was approved by the Ethics and Anthropology Committee of the Affiliated Cancer Hospital of Guangxi Medical University. All experiments and methods were performed in accordance with relevant guidelines and regulations.

### Immunohistochemical staining

All cancer specimens were immersed in formalin. Before staining, tissues were cut to 5 μm thickness and placed on glass slides. Endogenous peroxidase activity was inhibited and blocked by de-paraffinizing, rehydrating, and using 5% bovine serum albumin at 37°C for 30 min. The treated sections were incubated with anti-AIM2 (Signalway Antibody LLC, #36253) at 4°C overnight and washed three times with PBS. After that, incubation with secondary anti-peroxidation sunflower at 37°C for 30 minutes is required. After washing three times again with PBS, the sections were developed in diaminobenzidine and microscopic images were made by light microscopy.

### Data collection

The UCSC XENA database was used to download the TCGA and GTEx RNA expression spectra and clinical data, respectively (25). The TCGA database contains a total of 33 common tumors, please refer to **Supplementary Table 1** for the full names and abbreviations of the tumors. Copy number variation (CNV) data and methylation data were gathered from the TCGA. We used the GTEx database to analyze gene performance in normal tissues (26).

### Survival analysis

We performed univariate Cox regression analysis and Kaplan-Meier survival analysis using the R package. Univariate Cox regression analysis was used to assess the correlation of the expression of AIM2 inflammasomes with overall survival (OS), disease-specific survival (DSS), and progression-free interval (PFI) (25). Kaplan-Meier survival profiles were used to analyze and contrast disparities in survival time (27).

### Immune cell infiltration analysis

We analyzed the correlation of AIM2 inflammasomes with tumor-infiltrating immune cells, immune-activating genes, immune suppressor genes, chemokines, and chemokine receptors using single-sample gene set enrichment analysis (ssGSEA). Immune scores represented the infiltration of immune cells in tumor tissues.

### Methylation analysis

DNA methylation maps of patients were downloaded from the TCGA database (27). Methylation datasets of matched tumors and normal specimens from 14 cancer types were studied. The spearman’s correlation coefficient was used to compute the correlation of paired mRNA expression with the methylation dataset, which was then subjected to the t-distribution test. False discovery rate (FDR) adjusted p-values were used to identify genes with an FDR ≤0.05. Cox regression was implemented to estimate the hazard (risk of death). The hypermethylation group exhibited worse survival if the hazard ratio was >0. Hyper-worse was classified as high risk; otherwise, it was considered low risk (26).

### Pathway exploration for AIM2 inflammasomes at the single-cell level

To get further insight into the function and pathways of the AIM2 inflammasome in pan-cancer, we searched different databases. The Cancer Single-Cell State Atlas (CancerSEA) database (http://biocc.hrbmu.edu.cn/CancerSEA/) provides diverse functional states of specific genes at the single-cell level in different cancer types, allowing researchers to bypass the limitation of tumor heterogeneity. Correlations between AIM2 inflammasomes and functional states in various cancers were performed based on the CancerSEA database.

### Evaluation of the AIM2 inflammasomes score

The AIM2 inflammasomes score was calculated based on ssGSEA using the AIM2 inflammasome-related gene set, which was downloaded from the GSEA database (AIM2_INFLAMMASOME_COMPLEX), to quantify the expression levels of these genes for each cancer. R software gene set variation analysis package was used to perform the ssGSEA, which calculated the enrichment score of a gene set in a given sample.

### TIDE score analysis of AIM2 inflammasomes

A potential immune checkpoint blockade (ICB) response was predicted using the TIDE algorithm. TIDE utilizes a suite of gene expression markers to evaluate two separate mechanisms of tumor immune evasion, including dysfunction of tumor-infiltrating cytotoxic T lymphocytes (CTL) and rejection of CTL by immunosuppressive factors. Patients with high TIDE scores show a greater chance of tumor immune escape, and therefore, have a lower reaction rate to ICB treatment.

### Drug sensitivity analysis

The small molecules (n = 265), cell lines(n=860) and genes(n=17419) were collected from the Genomics of Drug Sensitivity in Cancer (GDSC) database. Analysis of the correlations among gene expression and drug sensitivity according to the method employed by Rees et al. (28). The half maximal inhibitory concentration (IC50) values for drugs and gene expression profiles for all cancer cell lines were downloaded from the GDSC. The Spearman’s correlation coefficients of the transcript levels and IC50 were calculated.

### Statistical analysis

Gene expression associations were estimated using Spearman’s correlation test. Student ‘s T test was used to compare the gene expression differences between the two groups. Kaplan-Meier survival curve was utilized to assess the prognostic significance of the indexes. Cox proportional risk models were applied to compute adjusted risk ratios. P < 0.05 was considered statistically significant (26).

## Result

### Differential expression of AIM2-related genes in pan-cancer and its impact on prognosis

**Figure 1:**
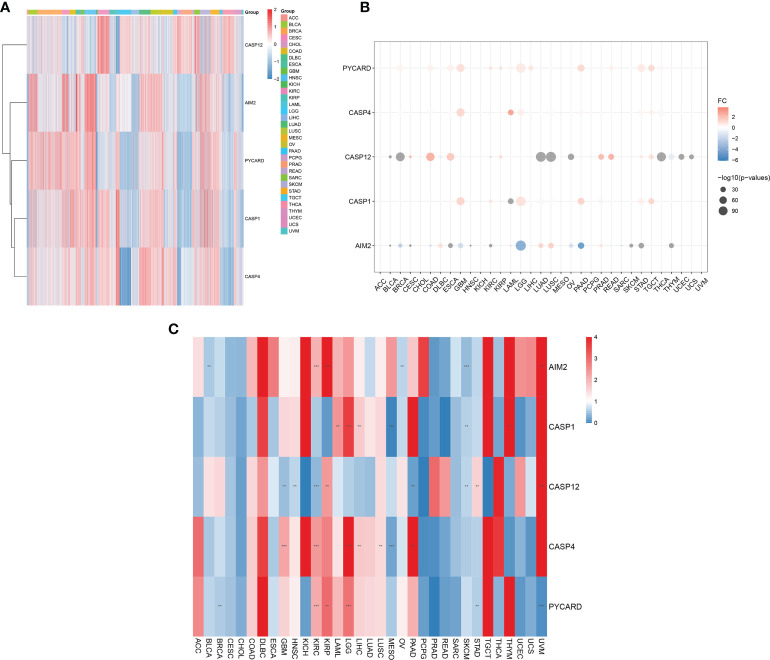
Differential expression of AIM2 inflammasome-related genes (AIM2i-RGs) in pan-cancer and its impact on prognosis. **(A)** Expression analysis of AIM2i-RGs in 33 tumors. Red represents high expression and blue represents low expression. **(B)** Differential expression analysis of AIM2i-RGs. Red indicates high expression in the tumor, blue indicates low expression in the tumor, and the larger the circle the smaller the P-value (Student’s t-test). **(C)** Analysis of the prognostic effect of the expression of AIM2i-RGs. Blue indicates Hazard Rate (HR) <1 and red indicates HR >1. (**p < 0.01, ***p < 0.001; COX regression).

We first assessed the expression of AIM2i-RGs in tumors and found that the expression of AIM2i-RGs differed in different tumors, suggesting that the expression pattern of AIM2i-RGs is tumor-specific (**Figure 1A**).

In a differential expression analysis of AIM2i-RGs in 33 tumors, we found that PYCARD had significantly higher expression in Bladder Urothelial Carcinoma (BLCA), Breast invasive carcinoma (BRCA), Cervical squamous cell carcinoma and endocervical adenocarcinoma (CESC), Colon adenocarcinoma (COAD), Esophageal carcinoma (ESCA), Glioblastoma multiforme (GBM), KIRC, Kidney renal papillary cell carcinoma (KIRP), Acute Myeloid Leukemia (LAML), Brain Lower Grade Glioma (LGG), Liver hepatocellular carcinoma (LIHC), Pancreatic adenocarcinoma (PAAD), Rectum adenocarcinoma (READ), Stomach adenocarcinoma (STAD), Testicular Germ Cell Tumors(TGCT), Thyroid carcinoma(THCA), and Uterine Corpus Endometrial Carcinoma(UCEC) (all P < 0.05). In contrast, CASP12 had significantly higher expression in CESC, COAD, ESCA, KIRP, Prostate adenocarcinoma (PRAD), READ, Skin Cutaneous Melanoma (SKCM), STAD, and TGCT and lower expression in BLCA, BRCA, Lymphoid Neoplasm Diffuse Large B-cell Lymphoma (DLBC), KIRC, Lung adenocarcinoma (LUAD), Lung squamous cell carcinoma (LUSC), Ovarian serous cystadenocarcinoma (OV), PAAD, THCA, Thymoma(THYM), UCEC, and Uterine Carcinosarcoma(UCS) (all P < 0.05). AIM2 had significantly higher expression in DLBC, LAML, LUAD, LUSC, and PRAD and lower in BLCA, BRCA, CESC, COAD, ESCA, GBM, Head and Neck squamous cell carcinoma (HNSC), KIRC, LGG, OV, PAAD, SKCM, STAD, and THYM (all P < 0.05) (**Figure 1B**). In our analysis of the prognostic effect of the expression of AIM2i-RGs, we found that: High expression of the PYCARD showed a good prognosis in BRCA, STAD, and Uveal Melanoma(UVM)and was a protective factor for patients. High expression of the PYCARD showed a bad prognosis in KIRC, KIRP, and LGG and was a hazard factor for patients. High expression of CASP12 showed a good prognosis in GBM, HNSC, KIRC, PAAD, and SKCM. High expression of CASP12 showed a bad prognosis in KIRP, STAD, and UVM. High expression of the AIM2 showed a good prognosis in BLCA, OV, and SKCM. High expression of the AIM2 showed a bad prognosis in KIRC, KIRP, and UVM. High expression of CASP1 showed a good prognosis in MESO and STAD. High expression of CASP1 showed a bad prognosis in LAML, LGG, LIHC and THYM. High expression of CASP4 showed a good prognosis in MESO. High expression of CASP1 showed a bad prognosis in GBM, KIRC, LGG, LIHC, LUSC, PAAD (**Figure 1C**, all P < 0.05).

### Analysis of AIM2i-RGs and gene mutation

**Figure 2:**
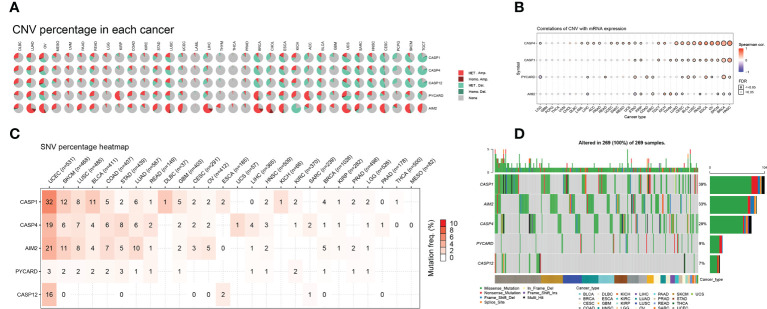
Distribution of copy number variation (CNV) pie charts for AIM2 inflammasome-related genes (AIM2i-RGs) with oncogene variant analysis in 33 tumors **(A)** Each CNV pie chart represents the CNV combinations of homozygous/heterozygotes of each AIM2i-RGs in each tumor. A single pie represents the proportional distribution of the different types of CNVs for a single gene in single cancer, with different colors representing the different types of CNVs. Heterozygous Amplification and Heterozygous Amplification; Homozygous Amplification and Homozygous Amplification; Heterozygous Deletion and Heterozygous Deletion; Homozygous Deletion and Homozygous Deletion; None, no CNV. **(B)** Correlation of CNV with mRNA expression. The size of the points represents statistical significance; the larger the point, the higher the statistical significance (Spearman’s correlation test). **(C)** Single nucleotide variant (SNV) frequencies of AIM2i-RGs in 26 tumors. Color shades represent the magnitude of mutation frequencies. The number size represents the frequency of samples with the corresponding mutated gene for particular cancer. No number indicates that no mutation has occurred in any region of the gene, and “0” indicates that no mutation has occurred in the coding region of the gene. **(D)** SNV Oncoplot. The side and top bar graphs show the number of variants in each sample or gene.

We further investigated the genetic variation analysis of AIM2i-RGs in 33 different tumors and found that they were associated with CNV in the vast majority of tumors. For the CASP12, Het Amplification was the predominant type of CNV in DLBC, UVM, LGG, and Kidney Chromophobe (KICH). Het Deletion was the predominant type of CNV in READ, STAD, LUSC, UCEC, LIHC, THYM, BRCA, Cholangiocarcinoma (CHOL), ESCA, Adrenocortical carcinoma (ACC), BLCA, GBM, UCS, Sarcoma (SARC), HNSC, CESC, Pheochromocytoma and Paraganglioma (PCPG), SKCM, and TGCT. For the AIM2, Het Amplification was the predominant type of CNV in the majority of tumors, and Het Deletion was the predominant type of CNV in the AIM2 only in KICH (**Figure 2A**).

We analyzed the correlation between the expression levels of AIM2i-RGs and CNV. The expression of CASP1 was positively correlated with CNV in STAD, KIRP, TGCT, THYM, LUAD, CESC, BLCA, LUSC, PAAD, ESCA, OV, SKCM, BRCA, and HNSC (**Figure 2B**, all p < 0.05). The expression of AIM2 was positively correlated with CNV in KIRP, KIRC, KICH, THYM, and CESC and negatively correlated with CNV in LIHC, STAD, LUAD, PAAD, SKCM, BRCA, and HNSC (**Figure 2B**, all p < 0.05). The above results show that CNV was one of the factors contributing to the elevated expression of the genes mentioned above.

We analyzed the mutations and variant types of AIM2i-RGs in each tumor (**Figure 2C**) and found that among the five genes, CASP1 had a higher percentage of single nucleotide variants (SNVs) in UCEC (32%). AIM2 had a higher percentage of SNVs in UCEC, SKCM, LUSC, COAD, STAD, LUAD, and OV, with AIM2 accounting for the highest proportion of SNVs in UCEC (21%). CASP4 had a higher percentage of SNVs in UCEC, SKCM, LUSC, COAD, STAD, and LUAD, with CASP4 accounting for the highest proportion of SNVs in UCEC (19%). Generally, PYCARD had a low proportion of SNV in the pan-cancer gene variants, with a maximum of 3% SNV in UCEC (**Figure 2C**). Missense mutations were the most common type of singly nucleotide polymorphisms (SNPs) for the above five genes in pan-cancer (**Figure 2D**), with CASP1 accounting for the highest percentage of SNPs at 39%. The remaining genes were AIM2 (33%), CASP4 (29%), PYCARD (9%), and CASP12 (7%) (**Figure 2D**).

### Differential analysis of methylation of AIM2i-RGs in pan-cancer

**Figure 3:**
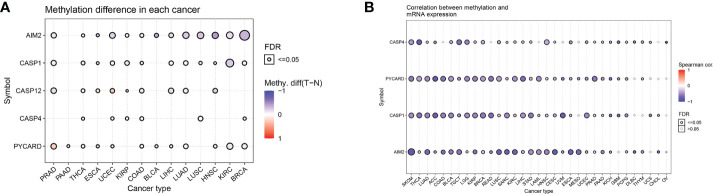
Differential methylation analysis of AIM2 inflammasome-related genes in pan-cancer **(A)** Differential methylation of AIM2i-RGs in 14 tumors. The different shades of color indicate the magnitude of the methylation levels, with red dots representing increased methylation in tumors and blue dots representing decreased methylation in tumors (Student’s t-test). **(B)** Correlation of methylation with the mRNA gene expression. The different shades of color indicate the magnitude of the correlation, with red dots representing positive correlation and blue dots representing negative correlation (Spearman’s correlation test).

Changes in DNA methylation have been observed in a variety of cancers, and they are thought to contribute to carcinogenesis. We analyzed the differential methylation of AIM2i-RGs in 14 tumors to better understand the mechanisms by which AIM2i-RGs affect tumorigenesis. We found that among the 14 tumors, AIM2 had low methylation levels in 12 tumors (PRAD, ESCA, UCEC, KIRP, COAD, BLCA, LIHC, LUAD, LUSC, HNSC, KIPC, and BRCA), on the other hand, AIM2 had high methylation levels in only one tumor (THCA) (all P < 0.05). PYCARD had high methylation levels in PRAD, THCA, UCEC, COAD, BLCA, and LUAD, and low methylation levels in PAAD, ESCA, LIHC, HNSC, KIRC, and BRCA (all P < 0.05) (**Figure 3A**). Meanwhile, by analyzing the correlation between the expression of AIM2i-RGs and methylation in 33 tumors, we also found that the expression of AIM2 was negatively correlated with methylation in SKCM, THCA, LUAD, ACC, COAD, BLCA, TGCT, LGG, KIRP, BRCA, Rectum adenocarcinoma Esophageal carcinoma (READ), LUSC, SARC, KIRC, LIHC, STAD, LAML, HNSC, CESC, UVM, ESCA, Mesothelioma (MESO), UCEC, PRAD, PAAD, KICH, PCPG, Lymphoid Neoplasm Diffuse Large B-cell Lymphoma (DLBC), THYM, and CHOL (all P < 0.05). The expression of PYCARD was negatively correlated with methylation in SKCM, THCA, LUAD, ACC, COAD, BLCA, TGCT, LGG, KIRP, BRCA, READ, LUSC, SARC, KIRC, LIHC, STAD, LAML, HNSC, CESC, UVM, ESCA, MESO, UCEC, PRAD, PAAD, KICH, GBM, PCPG, and THYM (all P < 0.05). The expression of CASP1 was negatively correlated with the methylation levels in SKCM, THCA, LUAD, ACC, COAD, BLCA, TGCT, LGG, KIRP, BRCA, READ, LUSC, SARC, KIRC, LIHC, STAD, LAML, HNSC, CESC, UVM, ESCA, UCEC, PRAD, PAAD, KICH, GBM, PCPG, UCS, and CHOL (all P < 0.05) (**Figure 3B**).

### Drug sensitivity analysis of AIM2i-RGs

**Figure 4:**
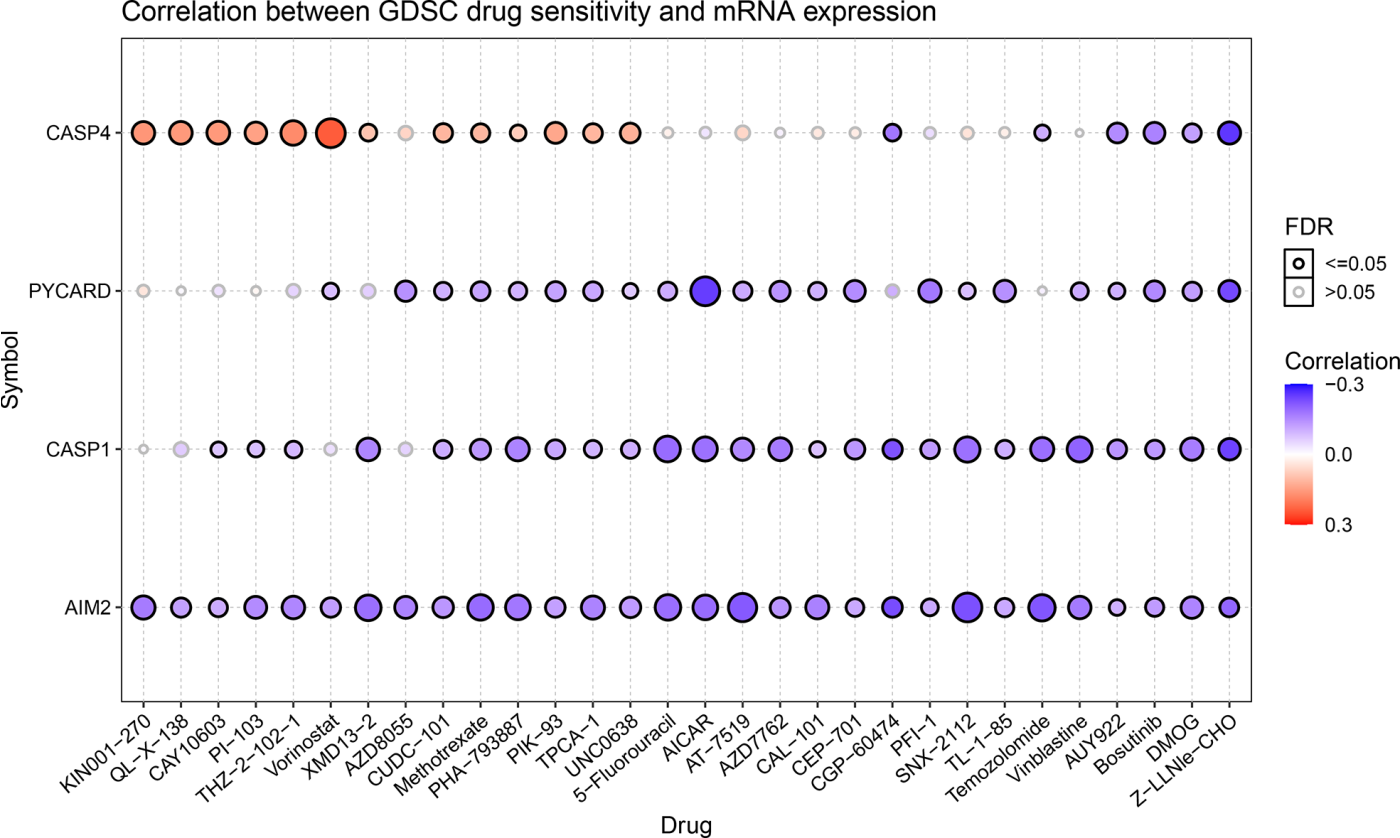
Drug sensitivity analysis of AIM2 inflammasome-related genes. Correlation of Genomics of Drug Sensitivity in Cancer(GDSC) drug sensitivity with mRNA expression. The shades of the color indicate the size of the correlation, red dots represent positive correlation and blue dots represent negative correlation. The size of the dots represents the statistical significance, the larger the dot, the higher the statistical significance (Spearman’s correlation test).

Genomic aberrations influence clinical response to therapy, which could be used as a potential biomarker for cancer drug screening. We integrated drug sensitivity (IC50) and gene expression profile data to better understand the role of AIM2i-RGs in chemotherapy or targeted therapy. Spearman’s correlation analysis showed that the expression of CASP4, PYCARD, CASP1, and AIM2 was negatively correlated with drug sensitivity to AUY922, Bosutinib, dimethyloxallyl glycine (DMOG), and Z-LNle-CHO (all P < 0.05) (**Figure 4**). These results suggest that modulating the expression of AIM2i-RGs may help improve sensitivity to chemotherapy and targeted drug therapy.

### Immunohistochemical validation of AIM2 expression

**Figure 5:**
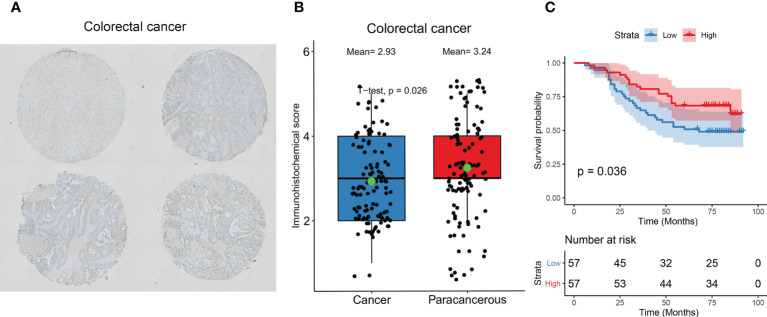
Immunohistochemical validation of AIM2 expression and prognosis. **(A)** Representative images of immunohistochemical detection of AIM2 expression in colorectal cancer. **(B)** Statistical results of AIM2 expression. (Green point represents mean values; Student’s t-test) **(C)** Survival analysis of AIM2. Patients were divided into high and low expression groups based on AIM2 expression (Log-rank test).

We examined the expression of AIM2 in 114 pairs of colorectal cancers versus paracancerous using immunohistochemistry and showed that AIM2 was significantly low expressed in colorectal cancers (**Figures 5A, B**, P=0.026). Survival analysis suggested that the OS of colorectal cancer patients in the low AIM2 group was significantly lower than that in the high expression group (**Figure 5C**, P=0.036), which was consistent with our analysis and previous reports (29–32).

### Differential expression of the AIM2 inflammasomes score and correlation with staging

**Figure 6:**
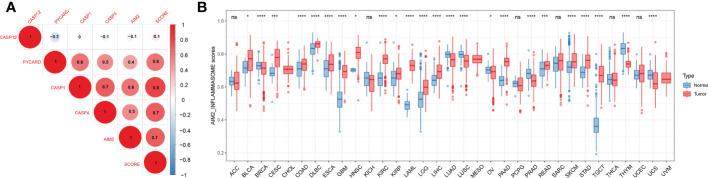
Differential expression of the AIM2 inflammasomes scores. **(A)** Correlation analysis of AIM2 inflammasome-related genes expression. The shades of color indicate the magnitude of the correlation, with red dots representing positive correlation and blue dots representing negative correlation. The size of the dots represents the correlation coefficient, the larger the dot, the stronger the correlation (Spearman’s correlation test). **(B)** AIM2 inflammasomes scores between tumor and normal samples for 33 cancers. *p < 0.05, ***p < 0.001, and ****p < 0.0001 ; ns, no statistical significance. (Student’s t-test).

We constructed an AIM2 inflammasomes score based on the ssGSEA algorithm to comprehensively assess the status of the AIM2 inflammasomes. We explored the association between the AIM2 inflammasomes score and AIM2i-RGs, and the results showed that the AIM2 inflammasomes score was significantly positively correlated with AIM2i-RGs (**Figure 6A**). Additionally, we observed significant strong correlations between AIM2i-RGs, suggesting a close association between the AIM2i-RGs (**Figure 6A**). In GTEx and TCGA, we estimated AIM2 inflammasomes scores between tumor and normal samples from 33 cancers. AIM2 inflammasomes scores were significantly increased in cancer samples than in normal samples, including in BLCA, CESC, COAD, DLBC, ESCA, GBM, HNSC, KIRC, KIRP, LAML, LGG, LIHC, OV, PAAD READ, SKCM, STAD, and TGCT (all P < 0.05). AIM2 inflammasomes scores were expressed at lower levels in BRCA, LUAD, LUSC, PRAD, THYM, and UCS than in normal samples (all P < 0.05) (**Figure 6B** **).** Most tumors had higher AIM2 inflammasomes scores than normal samples, suggesting that AIM2 inflammasomes may play a vital role in tumor development.

We further investigated the pan-cancer expression levels of the AIM2 inflammasomes scores at different stages. We found that AIM2 inflammasomes scores were significantly elevated in the early stages in COAD, HNSC, and LIHC (all P < 0.05). In contrast, AIM2 inflammasomes scores were only detected in KIRC with elevated scores in the late tumor stages (**Supplementary Figure 1**).

### Prognostic significance of the AIM2 inflammasomes score in tumors

**Figure 7:**
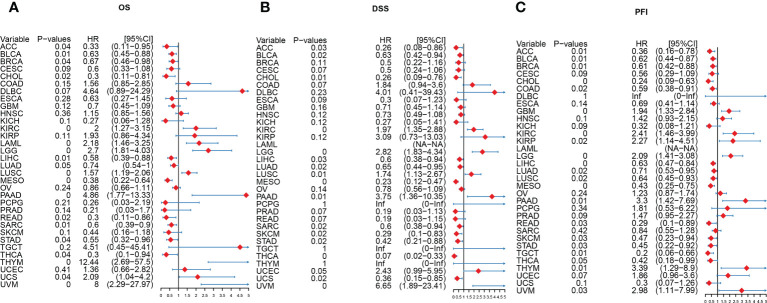
A Forest plot of the univariate Cox regression analysis of AIM2 inflammasomes. **(A)** OS is overall survival (COX regression). **(B)** DSS is disease-specific survival (COX regression). **(C)** PFI is the progression-free interval (COX regression).

To explore the correlation between the expression of AIM2 inflammasomes and tumor survival, we performed OS, DSS, and PFI analyses based on the AIM2 inflammasomes score. The results of Kaplan-Meier survival curves for AIM2 inflammasomes score in different tumors are shown in **Supplementary Figures 2**–**4** (all P < 0.05). We explored the prognostic significance of the AIM2 inflammasomes scores, using univariate Cox regression analysis. The OS results showed that high AIM2 inflammasomes scores showed a good prognosis in ACC, BLCA, BRCA, CHOL, LIHC, LUAD, MESO, READ, SARC, STAD, and THCA and were protective factors for patients. High AIM2 inflammasomes scores showed a bad prognosis in KIRC, LAML, LGG, LUSC, PAAD, THYM, UCS, and UVM and were hazard factors for patients (all P < 0.05) (**Figure 7A**). The DSS results showed that high AIM2 inflammasomes scores showed a good prognosis in ACC, BLCA, CHOL, LIHC, LUAD, MESO, SARC, SKCM, STAD, THCA, and UCS and were protective factors for patients. High AIM2 inflammasomes scores showed a bad prognosis in KIRC, LGG, LUSC, PAAD, UCEC, and UVM and were hazard factors for patients (all P < 0.05) (**Figure 7B**). The PFI results showed that high AIM2 inflammasomes scores showed a good prognosis in ACC, BLCA, BRCA, CHOL, COAD, LIHC, LUAD, LUSC, MESO, READ, SKCM, STAD, TGCT, and THCA and were protective factors for patients. High AIM2 inflammasomes scores showed a bad prognosis in GBM, KIRC, KIRP, LGG, PAAD, PRAD, THYM, and UVM and were hazard factors for patients (all P < 0.05) (**Figure 7C**). These results suggest that AIM2 inflammasomes scores have the potential to supplement traditional prognostic predictors and aid in the prediction of prognosis in patients with a wide range of tumors.

### Analysis of the function of the AIM2 inflammasomes score at the single-cell level

**Figure 8:**
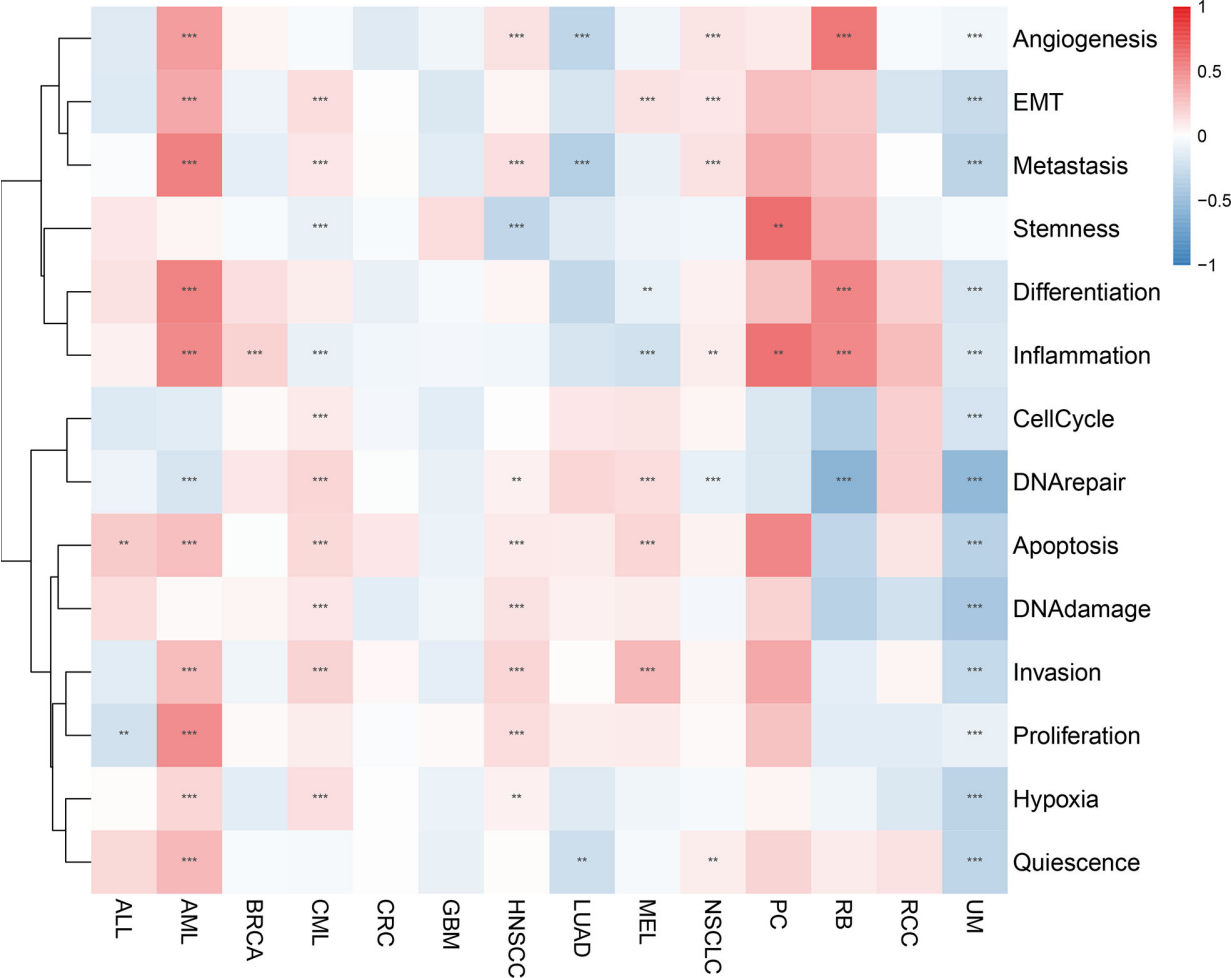
Correlation of the AIM2 inflammasomes scores with 14 functional status in different tumors. Red represents positive correlation and blue represents negative correlation. **p < 0.01, and ***p < 0.001 (Spearman’s correlation test).

We analyzed the correlation between AIM2 inflammasomes scores and 14 functional states in different tumors using CancerSEA data. The AIM2 inflammasomes scores were positively correlated with apoptosis in ALL, AML, CML, HNSCC, and MEL, and negatively correlated with apoptosis in UM (all P < 0.05). The AIM2 inflammasomes scores were positively correlated with inflammation in AML, BRCA, NSCLC, PC, and RB, and negatively correlated with inflammation in CML, MEL, and UM (all P < 0.05). The AIM2 inflammasomes scores were positively correlated with metastasis in AML, CML, HNSCC, and NSCLC, and negatively correlated with metastasis in LUAD and UM (all P < 0.05) (**Figure 8**).

### Correlation of the AIM2 inflammasomes score with tumor purity

**Figure 9:**
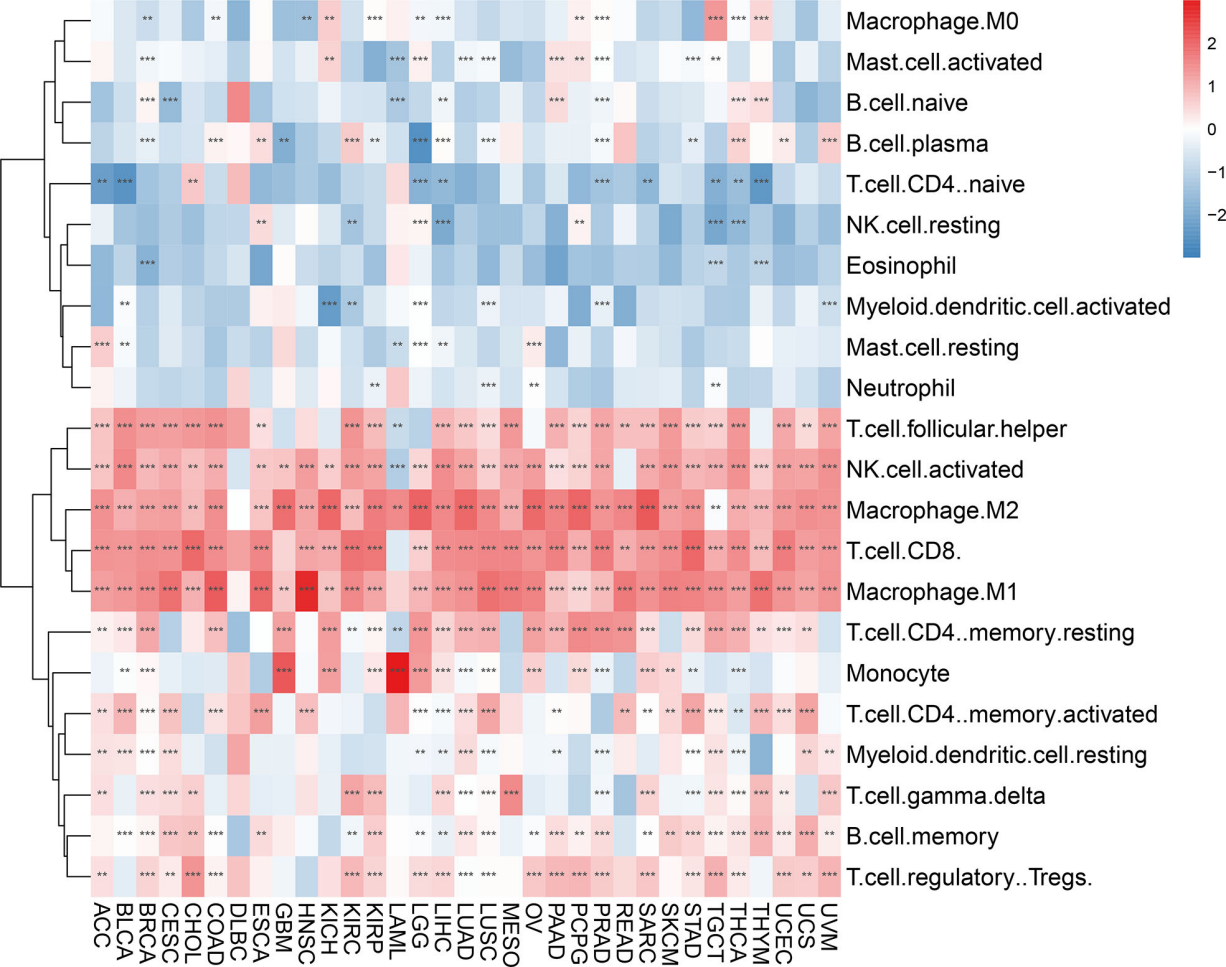
Immune cell infiltration analyses. Relevance of the AIM2 inflammasomes scores to tumor-infiltrating immune cells in 33 tumors. The red color indicates a positive correlation and the blue color indicates a negative correlation. **p < 0.01, and ***p < 0.001. (Spearman’s correlation test).

When we examined the correlation between AIM2 inflammasomes scores and tumor-infiltrating immune cells in 33 tumors, we found that AIM2 inflammasomes scores had a significant positive correlation with the levels of T cell follicular helper, natural killer (NK) cell activated, macrophage M2, T cell CD8, and macrophage M1 infiltration in most tumors. Additionally, the AIM2 inflammasomes score showed a negative correlation with T cell CD4 naïve infiltration levels in ACC, BLCA, LGG, LIHC, PRAD, SARC, TGCT, THCA, and THYM (all P < 0.05) (**Figure 9**).

**Figure 10:**
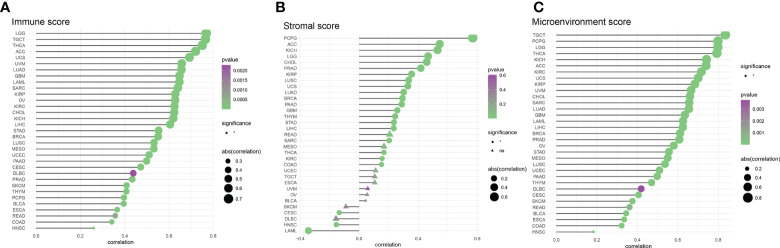
Correlation of AIM2 inflammasomes scores with tumor purity **(A)** Correlation of AIM2 inflammasomes scores with tumor Immune Score (Spearman’s correlation test). **(B)** Correlation of AIM2 inflammasomes scores with tumor Stromal Score (Spearman’s correlation test). **(C)** Correlation of AIM2 inflammasomes scores with tumor Microenvironment Score (Spearman’s correlation test) (all P < 0.05).

In an analysis of the tumor ImmuneScore, the AIM2 inflammasomes score was found to be positively correlated with the level of immune cell infiltration in GBM, PRAD, ESCA, CESC, MESO, OV, UCEC, PAAD, SARC, COAD, LUAD, BLCA, KIRP, BRCA, STAD, LUSC, TGCT, LIHC, THYM, SKCM CHOL, LAML, KICH, UVM, KIRC, READ, UCS, THCA, LGG, ACC, HNSC, DLBC, and PCPG (all P < 0.05) (**Figure 10A** and **Supplementary Figure 5**). In an analysis of the tumor StromalScore, the AIM2 inflammasomes score was positively correlated with the stromal cell score in PCPG, ACC, KICH, LGG, CHOL, PRAD, KIRP, LUSC, UCS, LUAD, BRCA, PAAD, GBM, THYM, STAD, LIHC, SARC, THCA, KIRC, and COAD (all P < 0.05). The AIM2 inflammasomes score was negatively correlated with stromal cell score in CESC, HNSC, and LAML (all P < 0.05) (**Figure 10B**). In the analysis of the tumor MicroenvironmentScore, the AIM2 inflammasomes score was positively correlated with the tumor microenvironment score in TGCT, PCPG, LGG, THCA, KICH, ACC, KIRC, UCS, KIRP, UVM, CHOL, SARC, LUAD, GBM, LAML, LIHC, BRCA, PRAD, OV, STAD, MESO, LUSC, UCEC, PAAD, THYM, DLBC, CESC, SKCM, READ, BLCA, ESCA, COAD, and HNSC (all P < 0.05) (**Figure 10C**).

### Correlation of the AIM2 inflammasomes score with immune-related genes

**Figure 11:**
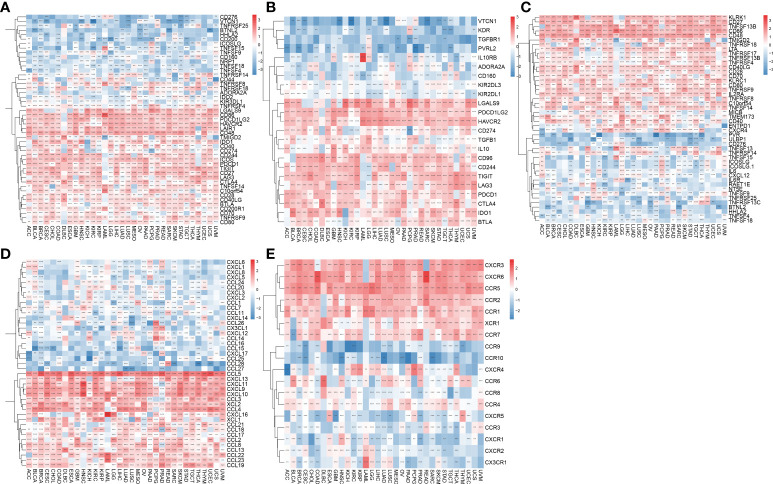
Association of AIM2 inflammasomes scores with immune-related genes. **(A)** Relevance between AIM2 inflammasomes scores and immune checkpoint in human tumors (Spearman’s correlation test). **(B)** Correlation between AIM2 inflammasomes scores and immunosuppressive genes in human tumors (Spearman’s correlation test). **(C)** Correlation between AIM2 inflammasomes scores and immune activation genes in human tumors (Spearman’s correlation test). **(D)** Correlation between AIM2 inflammasomes scores and chemokines in human tumors (Spearman’s correlation test). **(E)** Correlation between AIM2 inflammasomes scores and chemokine receptors in human tumors (Spearman’s correlation test). **p < 0.01, and ***p < 0.001.

Immune checkpoint genes have been shown to have a significant effect on immune cell infiltration and immunotherapy. Thereafter, we explored the association between AIM2 inflammasomes scores and immune checkpoint genes (ICG) in human tumors to explore the potential of AIM2 inflammasomes in immunotherapy. The results of the correlation between AIM2 inflammasomes scores and ICG showed that in the vast majority of tumors, AIM2 inflammasomes scores were positively correlated with the expression of LGALS9, CD86, PDCD1LG2, HAVCR2, LAIR1, CD48, ICOS, PDCD1, TIGIT, CD27, and LAG3, and negatively correlated with the expression of TNFSF9 (all P < 0.05) (**Figure 11A**). Additionally, we investigated the correlation between AIM2 inflammasomes scores and 23 immunosuppressive genes. The AIM2 inflammasomes score was positively correlated with the expression of LGALS9, PDCD1LG2, HAVCR2, CD96, CD244, TIGIT, LAG3, PDCD1, CTLA4, IDO1, and BTLA in the majority of tumors (all P < 0.05) (**Figure 11B**). The results of the correlation between AIM2 inflammasomes scores and 46 immune-activating genes in pan-cancer showed that the AIM2 inflammasomes score was positively correlated with KLRK1, CD27, TNFSF13B, CD86, CD48, TMIGD2, TNFRSF18, LTA, TNFRSF17, TNFRSF13B, TNFRSF4 CD40LG, CD28, CD70, KLRC1, CD80, TNFRSF9, IL2RA, TNFRSF8, C10orf54, TNFSF14, MICB, TMEM173, CD40, and ENTPD1 in the vast majority of tumors (all P < 0.05) (**Figure 11C**). Additionally, we investigated the correlation of AIM2 inflammasomes scores with chemokines and their receptor genes. The results showed that the AIM2 inflammasomes scores were positively correlated with the expression of CCL5, CXCL13, CXCL11, CXCL9, CXCL10, CCL3, XCL2, CCL4, XCL1, CCL21, CCL8, CCL13, CCL22, CCL23, and CCL19 chemokine genes in the vast majority of tumors, and negatively correlated with the expression of CCL11, CCL26, and CXCL2 (all P < 0.05) (**Figure 11D**). The AIM2 inflammasomes score was positively correlated with CXCR3, CXCR6, CCR5, CCR2, CCR1, XCR1, and CCR7 chemokine receptor genes and negatively correlated with CXCR1, CX3CR1, CCR3, and CCR8 in the vast majority of tumors (all P < 0.05) (**Figure 11E**).

### Correlation of the AIM2 inflammasomes score with the markers of immunotherapeutic response

**Figure 12:**
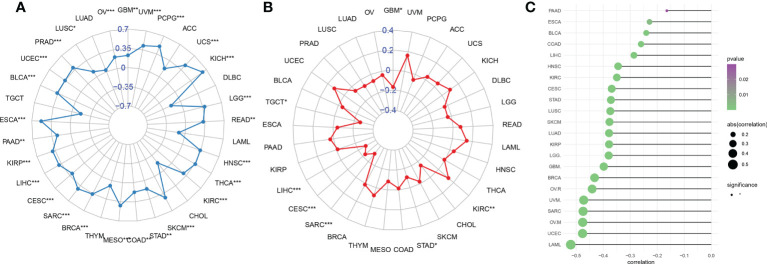
Relationship between AIM2 inflammasomes scores and markers of immunotherapeutic response in human cancers. **(A)** Correlation of AIM2 inflammasomes scores with tumor mutation burden in cancer (Spearman’s correlation test). **(B)** Correlation of AIM2 inflammasomes scores with microsatellite instability in cancer (Spearman’s correlation test). **(C)** Correlation of AIM2 inflammasomes scores with tumor immune dysfunction and exclusion scores. *p < 0.05, **p < 0.01, and ***p < 0.001 (Spearman’s correlation test).

Tumor immune escape can be monitored to predict the survival of patients with cancer treated with drugs. AIM2 inflammasomes scores were positively correlated with tumor mutation burden (TMB) in GBM, UVM, PCPG, UCS, KICH, LGG, READ, HNSC, THCA, KIRC, SKCM, STAD, COAD, MESO, BRCA, SARC, CESC, LIHC, KIRP, PAAD, ESCA, BLCA, UCEC, PRAD, LUSC, and OV (all P < 0.05) (**Figure 12A**). Thus, AIM2 inflammasomes scores were positively correlated with TMB in a majority of tumors. We also analyzed the correlation between AIM2 inflammasomes scores and microsatellite instability (MSI) with the following results. In KIRC, the AIM2 inflammasomes scores were positively correlated with MSI (all P < 0.05). The AIM2 inflammasomes scores were negatively correlated with MSI in GBM, STAD, SARC, CESC, LIHC, and TGCT (all P < 0.05) (**Figure 12B**). Currently, TIDE scores are the most promising marker of ICB response. TIDE scores have been reported to be more accurate than programmed death-1 (PD-L1) expression levels and TMB in predicting survival outcomes in cancer patients treated with ICB agents. Patients with high TIDE scores have a higher chance of tumor escape, therefore, they exhibit lower response rates to ICB therapy. In our analysis of TIDE and AIM2 inflammasomes scores for 22 tumors, we observed a negative correlation between all AIM2 inflammasomes scores and TIDE scores, which was significant in LAML, UCEC, OVM, SARC, UVM, OVR, and BRCA (**Figure 12C**) (**Supplementary Figure 6**). These results suggest that AIM2 inflammasomes may be associated with ICB response and may be a potential marker for ICB treatment.

### Single-cell transcriptome analysis of the AIM2 inflammasomes in the KIRC tumor microenvironment

**Figure 13:**
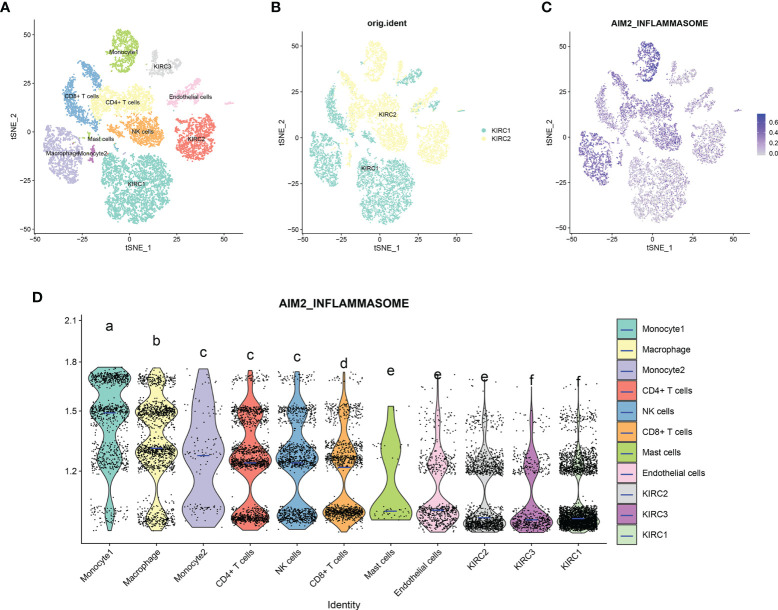
Single-cell transcriptomic atlas of Kidney renal clear cell carcinoma (KIRC). **(A)** A tSEN plot of KIRC samples with 11 distinct cell types. **(B)** A tSEN plot of KIRC from two different samples. **(C)** A tSEN plot of AIM2 inflammasomes scores in different cell types. **(D)** Comparison of AIM2 inflammasomes scores in different KIRC tumor microenvironment cells. The blue horizontal line on the violin plot indicates the median AIM2 inflammasomes score. The letters at the top indicate whether there is a statistical difference between the two cells. Different letters indicate that the difference is statistically significant (Student’s t-test).

scRNA-seq was performed on two in-house KIRC samples. Following QC using Seurat, 13124 high-quality single-cell transcriptome information was used for subsequent analysis. Cell clustering analysis based on the t-Distributed Stochastic Neighbor Embedding (tSNE) algorithm showed that the above cells could be classified into 11 clusters, namely KIRC1, KIRC2, KIRC3, monocyte1, monocyte2, macrophage, mast cells, endothelial cells, NK cells, CD4+ T cells, and CD8+ T cells (**Figure 13A**). Marker genes were significantly differentially expressed in different cell clusters (**Supplementary Figure 7**). Additionally, we found that tumor cells from two different sources of KIRC samples shared the same cluster (KIRC3) and unique clusters (KIRC1 and KIRC2) (**Figure 13B**). The above results suggest that KIRC cell types are heterogeneous. We used ssGSEA to impute AIM2 inflammasomes scores for KIRC tumor microenvironment cells and compared the differences in AIM2 inflammasomes scores across cell types (**Figure 13C**). Interestingly, we found significant differences in AIM2 inflammasomes scores across different cells (**Figure 13D**). Monocyte1 had significantly higher AIM2 inflammasomes scores than any of the other cells, suggesting AIM2 inflammasomes were significantly activated in the KIRC tumor microenvironment (P < 0.05). KIRC cells had the lowest AIM2 inflammasomes score, suggesting that the AIM2 inflammasomes reflect the tumor microenvironment rather than the tumor itself. The AIM2 inflammasomes scores differed significantly among the different KIRC cell clusters, suggesting that AIM2 inflammasomes may be a characteristic of KIRC cells. These results suggest that AIM2 inflammasomes are significantly different in different cells of the KIRC tumor microenvironment and that targeting AIM2 inflammasomes may be a breakthrough in regulating the tumor microenvironment.

## Discussion

We illustrate the process of this article by making a graphical abstract (**Supplementary Figure 8**).

AIM2 inflammasomes, the prototype and most characteristic member of ALR, induce caspase-1 activation by aggregating an adaptor protein called ASC (CARD-containing apoptosis-specific protein) after recognizing ds DNA. This activation leads to the maturation and release of the inflammatory cytokines such as interleukin (IL)-1β and pre-IL-18, resulting in activity proptosis (5). Extensive evidence suggests that AIM2 inflammasomes are correlated with tumor progression, treatment response and prognosis (14). Furthermore, AIM2 inflammasomes play a key role in preventing tumorigenesis by regulating host immunity (8). Previous studies have shown that AIM2 inflammasomes inhibit the occurrence and development of liver cancer (14), while promotes the occurrence and development of skin SCC (30). Therefore, that the role of AIM2 inflammasomes is variable, and AIM2 inflammasomes are a double-edged sword against cancer in that they can prevent or promote tumor development in depending on cancer type. Studying the application of AIM2 inflammasomes in different types of cancer may have important implications. Although studies on AIM2 inflammasomes have been specifically reported in some cancers, its relationship with immunity remains unclear. In this study, we analyzed the tumorigenic and prognostic value of AIM2 inflammasomes in various tumor types.

First, we analyzed the expression of AIM2i-RGs, gene changes, and immune invasion in the TCGA tumor types. We found that AIM2 inflammasomes were widely expressed in a variety of tissues and that their expression was upregulated in most tumors, with the expression levels of GBM, KIRC, and LAML being significantly upregulated.

The association between AIM2 inflammasomes and survival was assessed to further clarify the role of AIM2 inflammasomes in clinical risk stratification. Survival analysis showed that AIM2 overexpression was correlated with OS, DSS, and PFI. High expression of AIM2 is associated with poor prognosis of some tumor types, including KIRC, UVM, PAAD, and LGG. At the same time, previous studies have found that the loss of AIM2 expression has a close relationship with the low survival rate of patients with colorectal cancer (33). Compared with normal liver tissues, the relative expression levels of AIM2 mRNA and protein in liver cancer tissues were significantly downregulated, and their expression levels were significantly negatively associated with disease-related clinical indicators such as tumor volume, size, clinical stages, and pathological grade. Overexpression of AIM2 *in vitro* promoted the formation of the AIM2-ASC-Caspase 1 inflammatory vesicle complex and inhibited the activation of the mTORC1 signaling pathway through the activation of inflammasomes, which had oncogenic effects; on the contrary, inhibition of AIM2 expression promoted the deterioration of HCC (34).

Epigenetic changes in genes encoding inflammasome components often confer cancer susceptibility (35). Differential methylation analysis showed in the 10 AIM2i-RGs are hypomethylated, including BRCA, HNSC, etc. Thus, our findings could provide a basis for further studies on the methylation of AIM2 inflammasomes in tumorigenesis and progression. Previous studies have shown that AIM2 significantly inhibits the PI3K/AKT pathway, which has been shown to inhibit the development of colon tumors (36). Meanwhile, AIM2 inhibited colon cancer cell proliferation by inducing cell cycle arrest in the G2/M phase (37). Therefore, we can speculate that mutations in AIM2i-RGs may disrupt the gene replication cycle and function, and participate in the occurrence and development of tumors.

To better explore the association between AIM2i-RGs and clinical treatment, we studied the correlation between related genes and drugs and found that the expression level of AIM2i-RGs was correlated with the sensitivity of drugs, including AICAR, AT-7519, Bosutinib, DMOG, and Z-LLNLE-CHO. Previous studies have shown that *TET2*, plays a significant role in myeloid malignancies and inflammatory responses. TET2 was found to be strongly associated with the expression of AIM2 (38), and DMOG inhibits TET2 expression (39). Therefore, we can speculate that DMOG can regulate TET2 and affect the occurrence and development of myeloid malignancies by controlling the expression of AIM2i-RGs.

We further analyzed the association between AIM2 inflammasomes and tumor immunity. We assessed the correlation between common immune checkpoints and the expression of AIM2 inflammasomes. The strong correlation of AIM2 inflammasomes with several genes suggested that AIM2 inflammasomes may be a new target for tumor therapy. Previous studies have confirmed that TMB is positively correlated with tumor immunotherapy efficacy; the higher the tumor mutation load, the better is the efficacy of immune checkpoint inhibitors (40, 41). The expression of AIM2 inflammasomes in most tumors was positively related to TMB. Therefore, AIM2 inflammasomes was associated with increasing TMB, suggesting that AIM2 inflammasomes can be used to predict the curative effect of tumor immunotherapy. We found that the AIM2 inflammasomes score was significantly correlated with CXCR3, CXCR6, CCR5, CCR2, CCR1, CCR7, and other signaling molecules. Some studies show that CCL19 treatment could stronger the inhibiting effects of AIM2 overexpression on cell proliferation, migration, and invasion through CCR7. Therefore, CCL19 can activate the CCR7/AIM2 signaling pathway, suggesting that it could be a potential therapeutic method to treating gastric cancer (42).

The study has several limitations. This study only provides preliminary findings that AIM2 inflammasomes are associated with tumor progression in a variety of tumors, and more experimental work is needed to determine the precise molecular function and mechanisms of AIM2 inflammasomes in tumorigenesis. Further studies at cellular and molecular levels should be performed to substantiate our results. We lack direct evidence that AIM2 inflammasomes are involved in immune infiltration to influence prognosis. Meanwhile, the mechanisms by which AIM2 inflammasomes are involved in regulating immunity remains unclear. Additionally, we lack specific and complete cases with data to identify the function of the drugs in inhibiting tumor growth. Finally, of the five genes used in the construction of the AIM2 inflammasomes score, PYCARD, CASP1, CASP4 and CASP12 were not specific to AIM2 inflammasomes and may have an impact on the accuracy and specificity of the score. Further assessment is required after AIM2 inflammasomes-specific markers have been identified.

## Conclusion

In summary, we performed the first comprehensive analysis of AIM2 inflammasomes in pan-cancer and found that AIM2 inflammasomes are aberrantly expressed in a variety of methylated tumors. AIM2 inflammasomes are closely associated with tumor development and prognosis. Additionally, AIM2 inflammasomes are closely associated with immune checkpoints and regulatory genes. These results suggest that AIM2 inflammasomes seems to be very variable among cancer types and have the potential to become a new therapeutic target, therefore it must be investigated independently on each cancer type.

## Data availability statement

The datasets presented in this study can be found in online repositories. The names of the repository/repositories and accession number(s) can be found below: https://www.ncbi.nlm.nih.gov/geo/, GSE152938.

## Ethics statement

The studies involving human participants were reviewed and approved by the Ethics and Human Subject Committee of Guangxi Medical University Cancer Hospital. The patients/participants provided their written informed consent to participate in this study.

## Author contributions

YQ, LP, TQ, HR, YJZ, YZ, JL, WL, and JY: conceived and designed the experiments; YQ, LP, TQ, and HR: performed data collection; YJZ, YZ, WL, and JY: analyzed the data; YQ, LP, TQ, HR, YJZ, YZ, JL, WL, and JY: helped with the reagents/materials/analysis tools; YQ, LP, TQ, HR, YJZ, JL, YZ, WL, and JY: contributed to the writing of the manuscript. All authors contributed to the article and approved the submitted version.

## Funding

Guangxi Key R&D Program Project (2021AA19010); Self-financed Science and Technology Projects in Guangxi (Z-A20220144); Guangxi Major Special Projects (2021AB42022); Guangxi Multidisciplinary Collaborative Health Management Talent Mini-Highland (guizutongzi:2019-85).

## Conflict of interest

The authors declare that the research was conducted in the absence of any commercial or financial relationships that could be construed as a potential conflict of interest.

## Publisher’s note

All claims expressed in this article are solely those of the authors and do not necessarily represent those of their affiliated organizations, or those of the publisher, the editors and the reviewers. Any product that may be evaluated in this article, or claim that may be made by its manufacturer, is not guaranteed or endorsed by the publisher.

## Supplementary material

Supplementary Figure 1 Pan-cancer expression levels of the AIM2 inflammasomes score at different stages.

Supplementary Figure 2 Kaplan-Meier survival curves of disease-specific survival in patients with cancer with high versus low expressions of the AIM2 inflammasomes.

Supplementary Figure 3 Kaplan-Meier survival curves of overall survival in patients with cancer with high versus low expressions of the AIM2 inflammasomes.

Supplementary Figure 4 Kaplan-Meier survival curves of progression-free interval in patients with cancer with high versus low expressions of the AIM2 inflammasomes.

Supplementary Figure 5 Correlation of the AIM2 inflammasomes scores with the immune scores.

Supplementary Figure 6 Correlation of the AIM2 inflammasomes scores with the tumor immune dysfunction and exclusion scores.

Supplementary Figure 7 Expression of the marker genes in different cells in the Kidney renal clear cell carcinoma tumor microenvironment.

Supplementary Figure 8 The graphical abstract of the AIM2 inflammasomes.

## References

[1] Liu JN Kong XS Sun P Wang R Li W Chen QF. An integrated pan-cancer analysis of TFAP4 aberrations and the potential clinical implications for cancer immunity. J Cell Mol Med (2021) 25(4):2082–97. doi: 10.1111/jcmm.16147 PMC7882993 33373169

[2] Ribas A Wolchok JD. Cancer immunotherapy using checkpoint blockade. Science (2018) 359(6382):1350–5. doi: 10.1126/science.aar4060 PMC7391259 29567705

[3] Hegde PS Chen DS. Top 10 challenges in cancer immunotherapy. Immunity (2020) 52(1):17–35. doi: 10.1016/j.immuni.2019.12.011 31940268

[4] McAllister SS Weinberg RA. Tumor-host interactions: A far-reaching relationship. J Clin Oncol Off J Am Soc Clin Oncol (2010) 28(26):4022–8. doi: 10.1200/JCO.2010.28.4257 20644094

[5] Man SM Kanneganti TD. Regulation of inflammasome activation. Immunol Rev (2015) 265(1):6–21. doi: 10.1111/imr.12296 25879280 PMC4400844

[6] Ta A Vanaja SK. Inflammasome activation and evasion by bacterial pathogens. Curr Opin Immunol (2021) 68:125–33. doi: 10.1016/j.coi.2020.11.006 PMC7925435 33338767

[7] Alehashemi S Goldbach-Mansky R. Human autoinflammatory diseases mediated by NLRP3-, pyrin-, NLRP1-, and NLRC4-inflammasome dysregulation updates on diagnosis, treatment, and the respective roles of IL-1 and IL-18. Front Immunol (2020) 11:1840. doi: 10.3389/fimmu.2020.01840 32983099 PMC7477077

[8] Karki R Man SM Kanneganti TD. Inflammasomes and cancer. Cancer Immunol Res (2017) 5(2):94–9. doi: 10.1158/2326-6066.CIR-16-0269 PMC5593081 28093447

[9] Awad F Assrawi E Louvrier C Jumeau C Georgin-Lavialle S Grateau G. Inflammasome biology, molecular pathology and therapeutic implications. Pharmacol Ther (2018) 187:133–49. doi: 10.1016/j.pharmthera.2018.02.011 29466702

[10] Choubey D Walter S Geng Y Xin H. Cytoplasmic localization of the interferon-inducible protein that is encoded by the AIM2 (absent in melanoma) gene from the 200-gene family. FEBS Lett (2000) 474(1):38–42. doi: 10.1016/S0014-5793(00)01571-4 10828447

[11] Woerner SM Kloor M Schwitalle Y Youmans H Doeberitz M Gebert J. The putative tumor suppressor AIM2 is frequently affected by different genetic alterations in microsatellite unstable colon cancers. Genes Chromosomes Cancer (2007) 46(12):1080–9. doi: 10.1002/gcc.20493 17726700

[12] Farshchian M Nissinen L Siljamäki E Riihilä P Piipponen M Kivisaari A. Tumor cell-specific AIM2 regulates growth and invasion of cutaneous squamous cell carcinoma. Oncotarget (2017) 8(28):45825–36. doi: 10.18632/oncotarget.17573 PMC5542230 28526809

[13] Janowski AM Colegio OR Hornick EE McNiff JM Martin MD Badovinac VP. NLRC4 suppresses melanoma tumor progression independently of inflammasome activation. J Clin Invest (2016) 126(10):3917–28. doi: 10.1172/JCI86953 PMC5096827 27617861

[14] Chen SL Liu LL Lu SX Luo RZ Wang CH Wang H. HBx-mediated decrease of AIM2 contributes to hepatocellular carcinoma metastasis. Mol Oncol (2017) 11(9):1225–40. doi: 10.1002/1878-0261.12090 PMC5579341 28580773

[15] Martínez-Cardona C Lozano-Ruiz B Bachiller V Peiró G Algaba-Chueca F Gómez-Hurtado I. AIM2 deficiency reduces the development of hepatocellular carcinoma in mice. Int J Cancer (2018) 143(11):2997–3007. doi: 10.1002/ijc.31827 30133699

[16] de Koning HD van Vlijmen-Willems IM Zeeuwen PL Blokx WA Schalkwijk J. Absent in melanoma 2 is predominantly present in primary melanoma and primary squamous cell carcinoma, but largely absent in metastases of both tumors. J Am Acad Dermatol (2014) 71(5):1012–5. doi: 10.1016/j.jaad.2014.06.012 25437966

[17] DeYoung KL Ray ME Su YA Anzick SL Johnstone RW Trapani JA. Cloning a novel member of the human interferon-inducible gene family associated with control of tumorigenicity in a model of human melanoma. Oncogene (1997) 15(4):453–7. doi: 10.1038/sj.onc.1201206 9242382

[18] Zhang Z Dong X Yang X Wan D Sun L Gu M. Expression and clinical significance of absent in melanoma 2 in colorectal cancer. Biomedicine pharmacotherapy = Biomedecine pharmacotherapie (2017) 94:843–9. doi: 10.1016/j.biopha.2017.07.161 28802238

[19] Chai D Shan H Wang G Li H Fang L Song J. AIM2 is a potential therapeutic target in human renal carcinoma and suppresses its invasion and metastasis *via* enhancing autophagy induction. Exp Cell Res (2018) 370(2):561–70. doi: 10.1016/j.yexcr.2018.07.021 30031129

[20] Chai D Liu N Li H Wang G Song J Fang L. H1/pAIM2 nanoparticles exert anti-tumour effects that is associated with the inflammasome activation in renal carcinoma. J Cell Mol Med (2018) 22(11):5670–81. doi: 10.1111/jcmm.13842 PMC6201339 30160343

[21] Chen IF Ou-Yang F Hung JY Liu JC Wang H Wang SC. AIM2 suppresses human breast cancer cell proliferation *in vitro* and mammary tumor growth in a mouse model. Mol Cancer Ther (2006) 5(1):1–7. doi: 10.1158/1535-7163.MCT-05-0310 16432157

[22] Untergasser G Koch HB Menssen A Hermeking H. Characterization of epithelial senescence by serial analysis of gene expression: Identification of genes potentially involved in prostate cancer. Cancer Res (2002) 62(21):6255–62. https://aacrjournals.org/cancerres/article/62/21/6255/509349/Characterization-of-Epithelial-Senescence-by 12414655

[23] Xin H Curry J Johnstone RW Nickoloff BJ Choubey D. Role of IFI 16, a member of the interferon-inducible p200-protein family, in prostate epithelial cellular senescence. Oncogene (2003) 22(31):4831–40. doi: 10.1038/sj.onc.1206754 12894224

[24] Su C Lv Y Lu W Yu Z Ye Y Guo B. Single-cell RNA sequencing in multiple pathologic types of renal cell carcinoma revealed novel potential tumor-specific markers. Front Oncol (2021) 11:719564. doi: 10.3389/fonc.2021.719564 34722263 PMC8551404

[25] Zhou X Du J Liu C Zeng H Chen Y Liu L. A pan-cancer analysis of CD161, a potential new immune checkpoint. Front Immunol (2021) 12:688215. doi: 10.3389/fimmu.2021.688215 34305920 PMC8299557

[26] Zhang J Jiang H Du K Xie T Wang B Chen C. Pan-cancer analysis of genomic and prognostic characteristics associated with coronavirus disease 2019 regulators. Front Med (Lausanne) (2021) 8:662460. doi: 10.3389/fmed.2021.662460 34458283 PMC8385656

[27] Ju M Bi J Wei Q Jiang L Guan Q Zhang M. Pan-cancer analysis of NLRP3 inflammasome with potential implications in prognosis and immunotherapy in human cancer. Briefings Bioinf (2021) 22(4):1–16. doi: 10.1093/bib/bbaa345 PMC8294515 33212483

[28] Rees MG Seashore-Ludlow B Cheah JH Adams DJ Price EV Gill S. Correlating chemical sensitivity and basal gene expression reveals mechanism of action. Nat Chem Biol (2016) 12(2):109–16. doi: 10.1038/nchembio.1986 PMC4718762 26656090

[29] Man SM Zhu Q Zhu L Liu Z Karki R Malik A. Critical role for the DNA sensor AIM2 in stem cell proliferation and cancer. 162(1):45–58. doi: 10.1016/j.cell.2015.06.001 PMC4491002 26095253

[30] Ungerbäck J Belenki D Jawad ul-Hassan A Fredrikson M Fransén K Elander N. Genetic variation and alterations of genes involved in NFκB/TNFAIP3- and NLRP3-inflammasome signaling affect susceptibility and outcome of colorectal cancer. Carcinogenesis (2012) 33(11):2126–34. doi: 10.1093/carcin/bgs256 22843550

[31] Dihlmann S Tao S Echterdiek F Herpel E Jansen L Chang-Claude J. Lack of Absent in Melanoma 2 (AIM2) expression in tumor cells is closely associated with poor survival in colorectal cancer patients. Int J Cancer (2014) 135(10):2387–96. doi: 10.1002/ijc.28891 24729378

[32] Kim TM Laird PW Park PJ. The landscape of microsatellite instability in colorectal and endometrial cancer. Cell (2013) 155(4):858–68. doi: 10.1016/jcell201310015 PMC3871995 24209623

[33] Dihlmann S Tao S Echterdiek F Herpel E Jansen L Chang-Claude J. Lack of absent in melanoma 2 (AIM2) expression in tumor cells is closely associated with poor survival in colorectal cancer patients. Int J Cancer (2014) 135(10):2387–96. doi: 10.1002/ijc.28891 24729378

[34] Lozano-Ruiz B González-Navajas JM. The emerging relevance of AIM2 in liver disease. Int J Mol Sci (2020) 21(18):6535. doi: 10.3390/ijms21186535 PMC7555176 32906750

[35] Verma D Bivik C Farahani E Synnerstad I Fredrikson M Enerbäck C. Inflammasome polymorphisms confer susceptibility to sporadic malignant melanoma. Pigment Cell Melanoma Res (2012) 25(4):506–13. doi: 10.1111/j.1755-148X.2012.01008.x 22524199

[36] Wilson JE Petrucelli AS Chen L Koblansky AA Truax AD Oyama Y. Inflammasome-independent role of AIM2 in suppressing colon tumorigenesis *via* DNA-PK and akt. Nat Med (2015) 21(8):906–13. doi: 10.1038/nm.3908 PMC4529369 26107252

[37] Patsos G Germann A Gebert J Dihlmann S. Restoration of absent in melanoma 2 (AIM2) induces G2/M cell cycle arrest and promotes invasion of colorectal cancer cells. Int J Cancer (2010) 126(8):1838–49. doi: 10.1002/ijc.24905 19795419

[38] Guo X Zhong W Chen Y Zhang W Ren J Gao A. Benzene metabolites trigger pyroptosis and contribute to haematotoxicity *via* TET2 directly regulating the Aim2/Casp1 pathway. EBioMedicine (2019) 47:578–89. doi: 10.1016/j.ebiom.2019.08.056 PMC6796562 31474553

[39] Gao W Yu X Hao J Wang L Qi M Han L. Ascorbic acid improves parthenogenetic embryo development through TET proteins in mice. Biosci Rep (2019) 39(1):BSR20181730. doi: 10.1042/BSR20181730 30567727 PMC6328890

[40] Chalmers ZR Connelly CF Fabrizio D Gay L Ali SM Ennis R. Analysis of 100,000 human cancer genomes reveals the landscape of tumor mutational burden. Genome Med (2017) 9(1):34. doi: 10.1186/s13073-017-0424-2 28420421 PMC5395719

[41] Goodman AM Sokol ES Frampton GM Lippman SM Kurzrock R. Microsatellite-stable tumors with high mutational burden benefit from immunotherapy. Cancer Immunol Res (2019) 7(10):1570–3. doi: 10.1158/2326-6066.CIR-19-0149 PMC6774837 31405947

[42] Zhou R Sun J He C Huang C Yu H. CCL19 suppresses gastric cancer cell proliferation, migration, and invasion through the CCL19/CCR7/AIM2 pathway. Hum Cell (2020) 33(4):1120–32. doi: 10.1007/s13577-020-00375-1 32564199

