# Supplementary figures and images for: Pan-cancer analysis of AIM2 inflammasomes with potential implications for immunotherapy in human cancer: A bulk omics research and single cell sequencing validation
Source: Front Immunol. 2022 Sep 29;13:998266. doi: 10.3389/fimmu.2022.998266 (PMC9559585; doi:10.3389/fimmu.2022.998266)

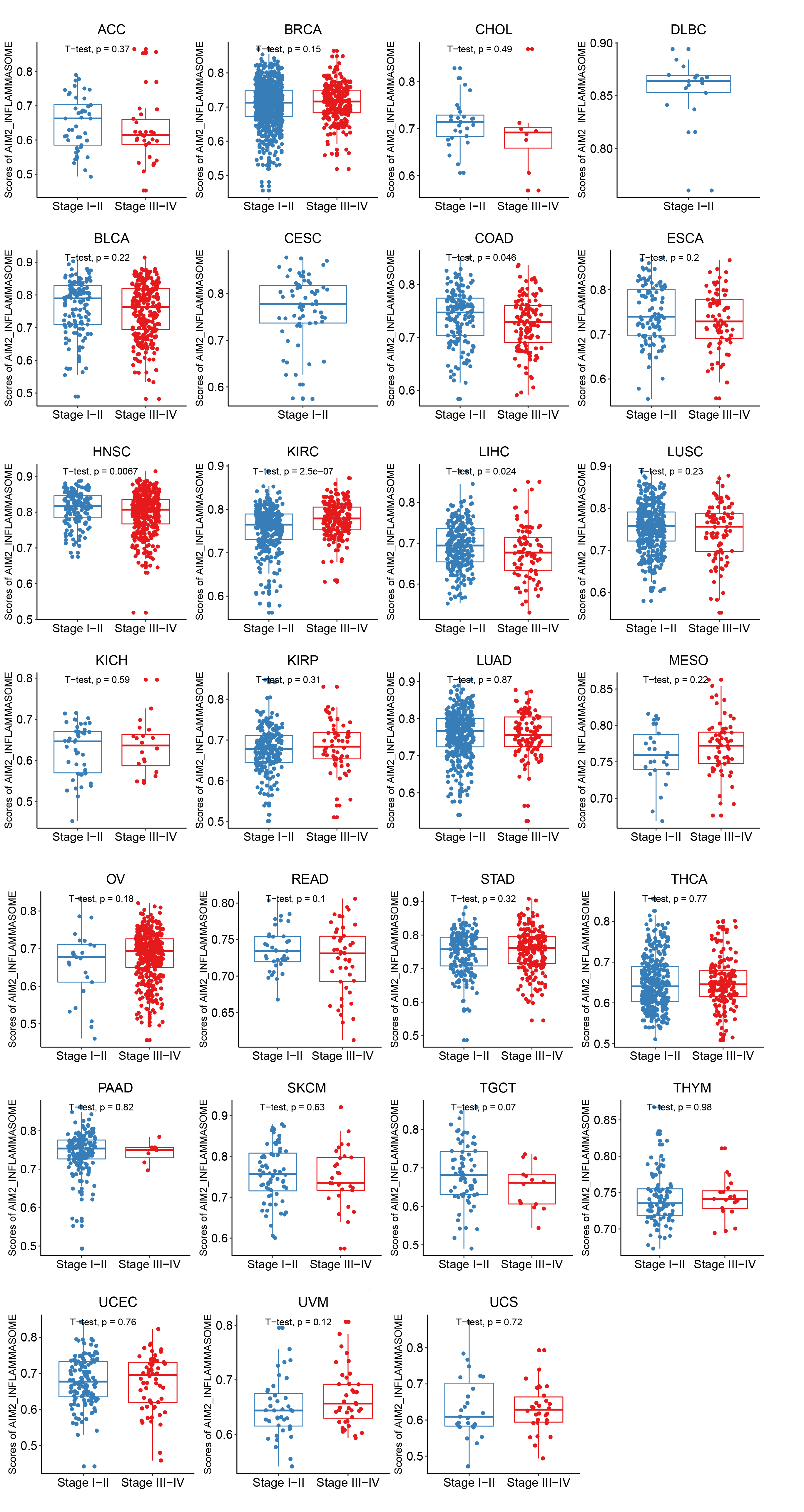

Supplement: Supplementary Figure 1 — Pan-cancer expression levels of the AIM2 inflammasomes score at different stages. [file Image_1.tif]

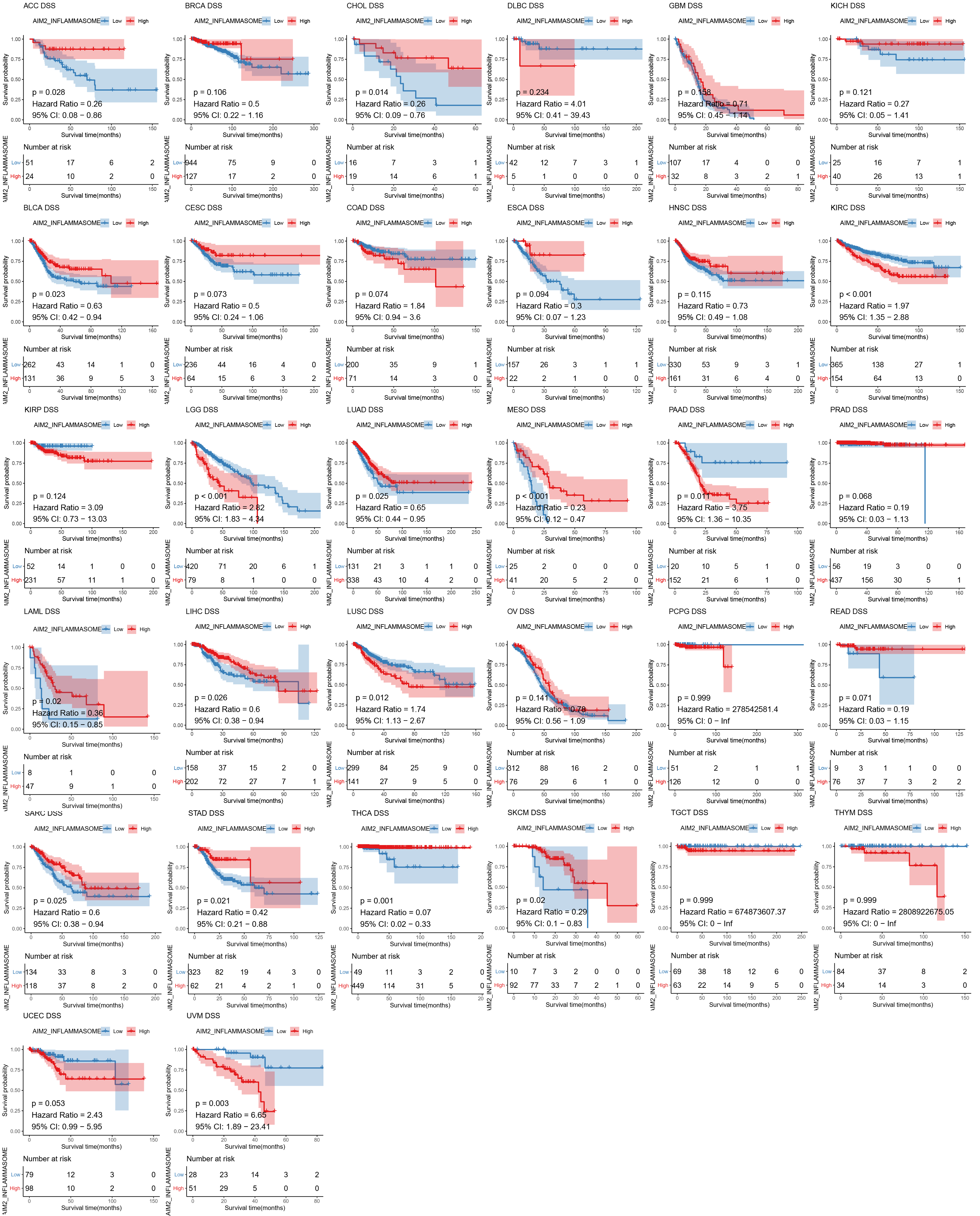

Supplement: Supplementary Figure 2 — Kaplan-Meier survival curves of disease-specific survival in patients with cancer with high versus low expressions of the AIM2 inflammasomes. [file Image_2.tif]

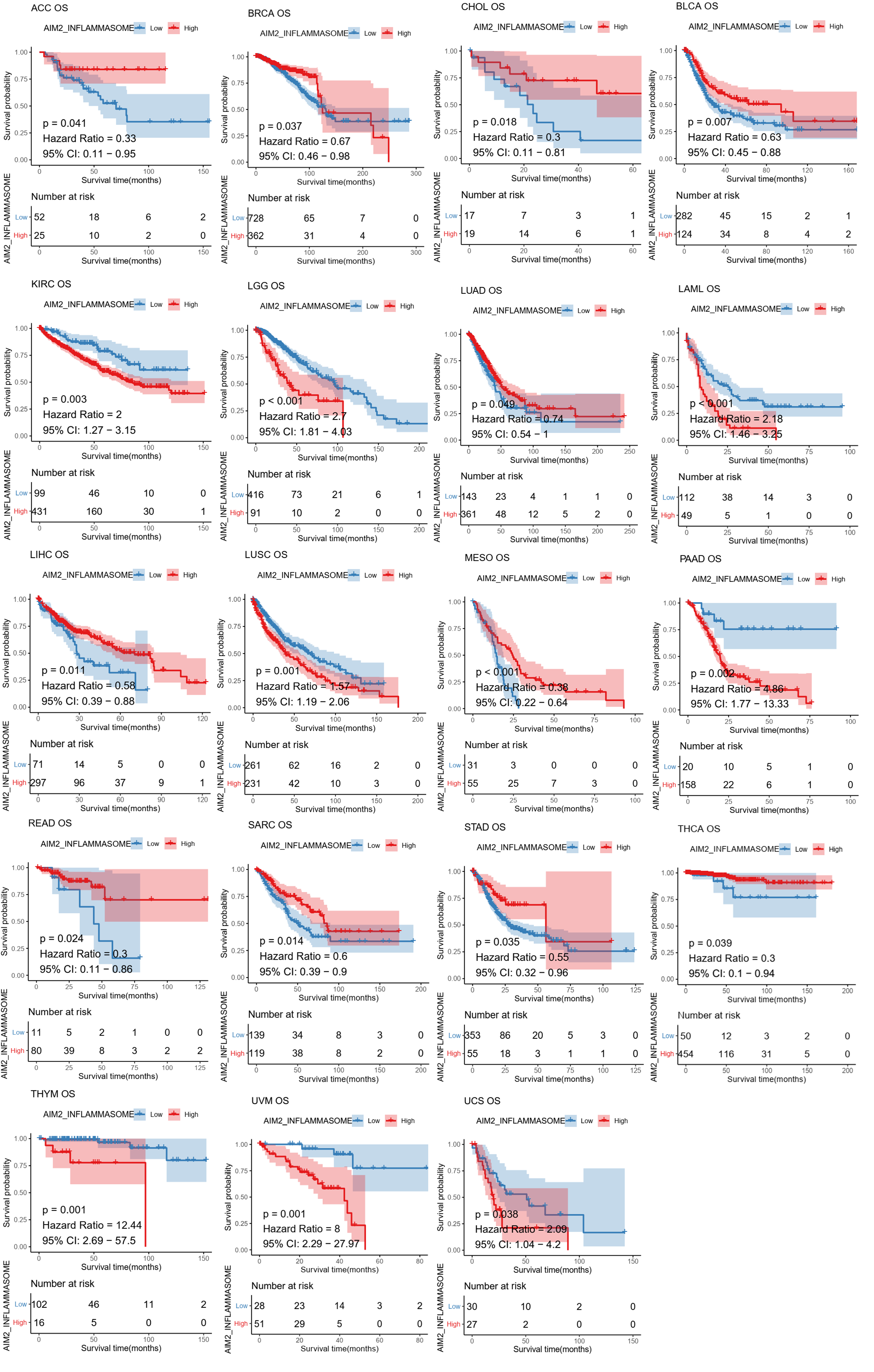

Supplement: Supplementary Figure 3 — Kaplan-Meier survival curves of overall survival in patients with cancer with high versus low expressions of the AIM2 inflammasomes. [file Image_3.tif]

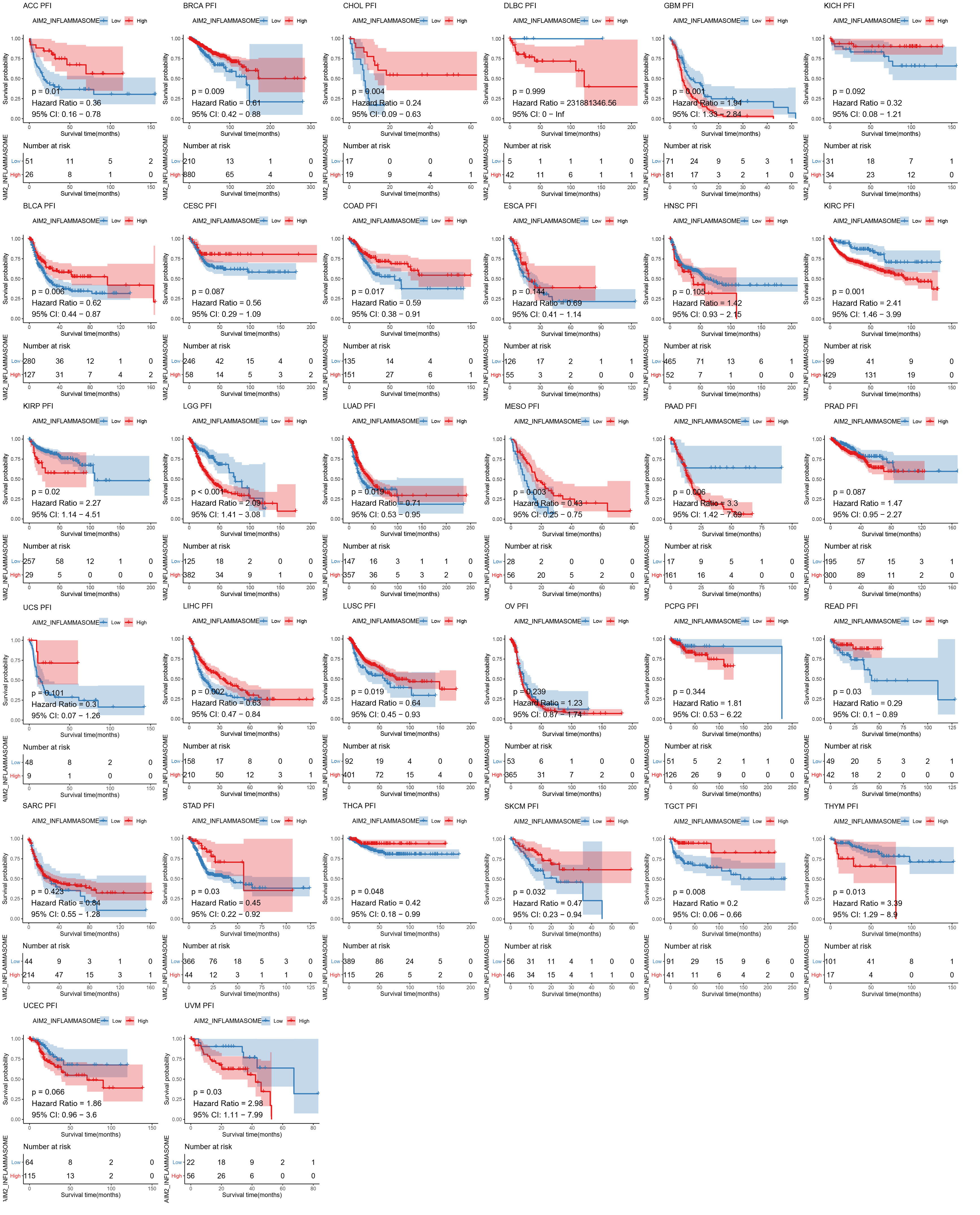

Supplement: Supplementary Figure 4 — Kaplan-Meier survival curves of progression-free interval in patients with cancer with high versus low expressions of the AIM2 inflammasomes. [file Image_4.tif]

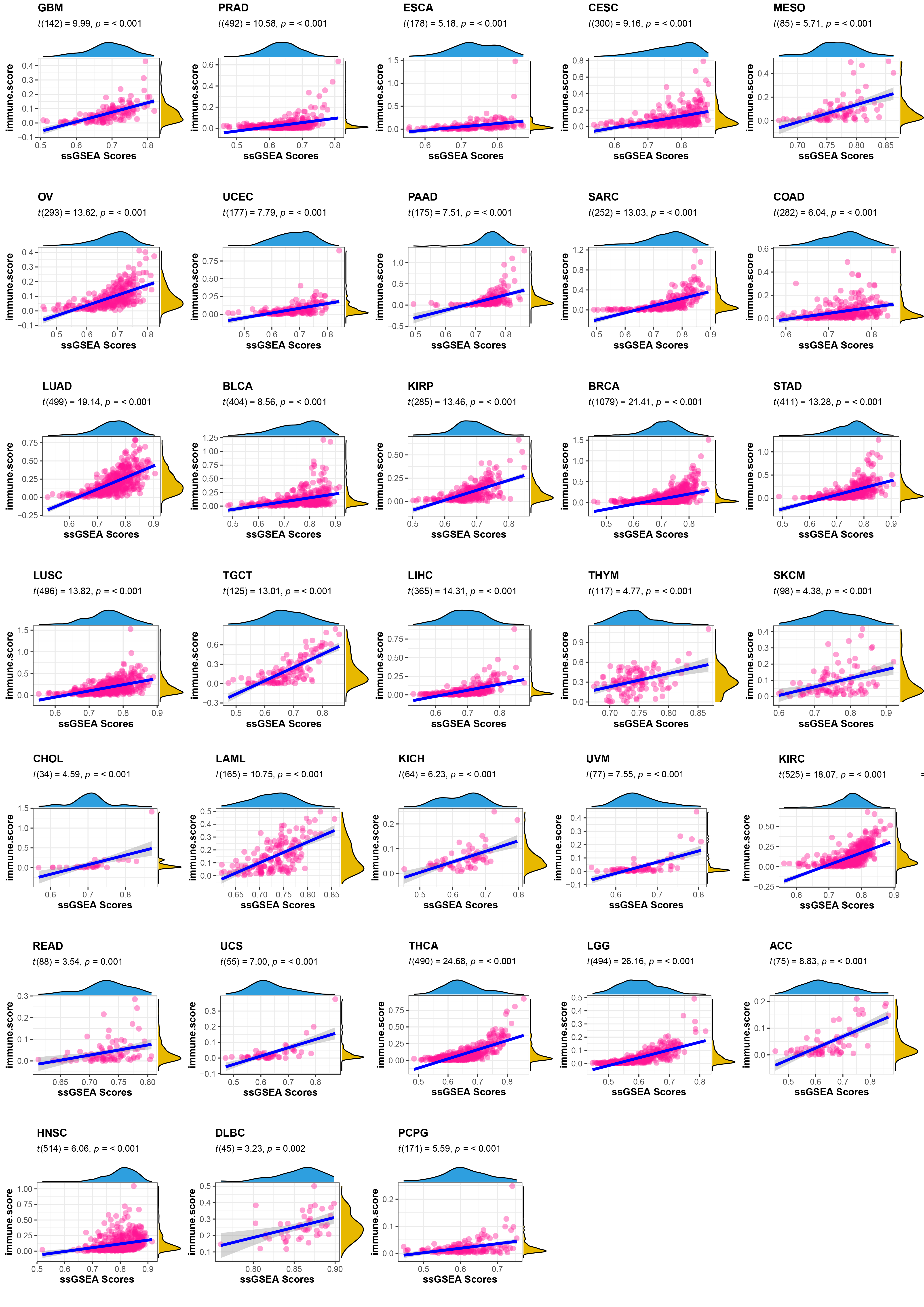

Supplement: Supplementary Figure 5 — Correlation of the AIM2 inflammasomes scores with the immune scores. [file Image_5.tif]

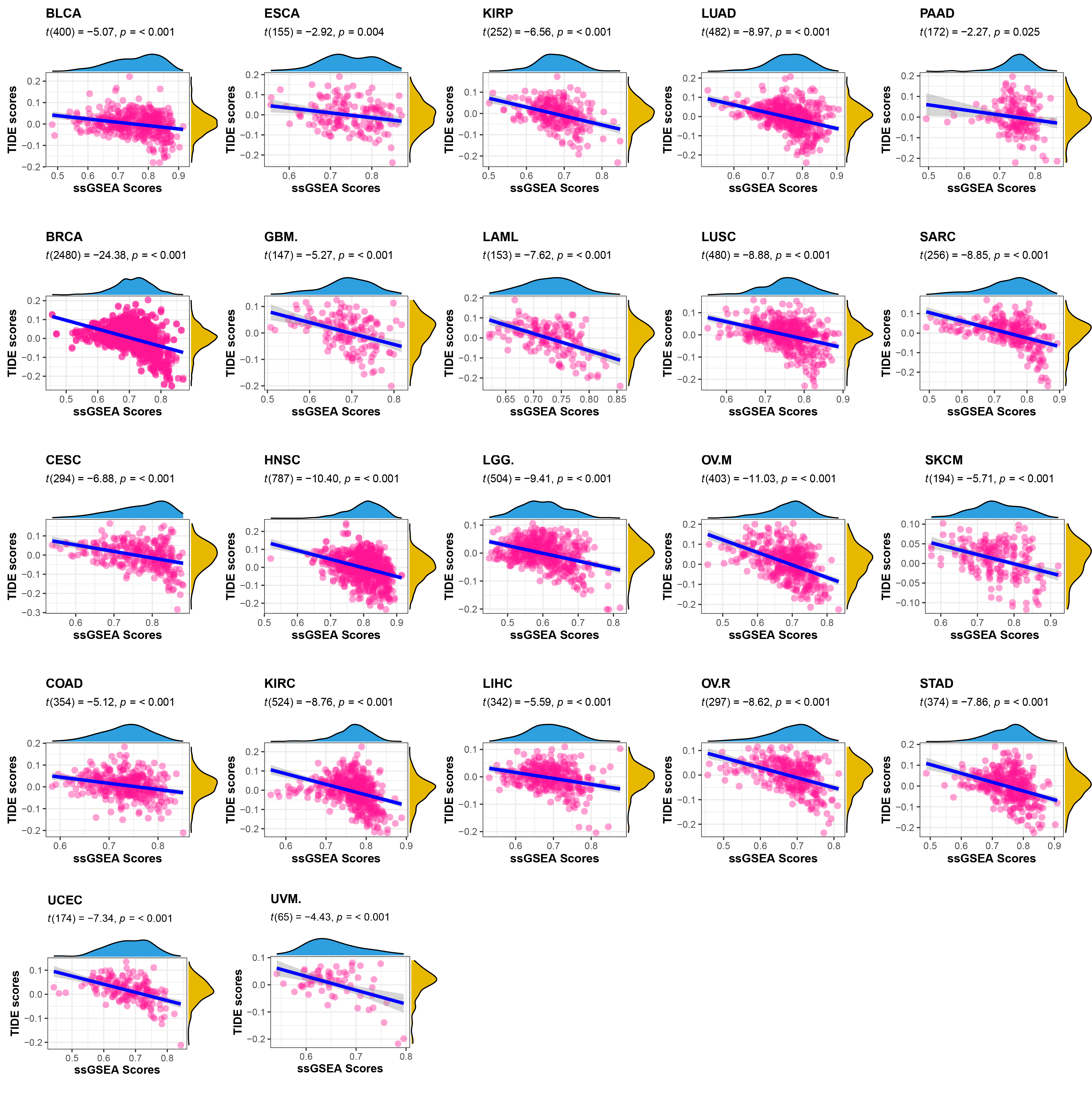

Supplement: Supplementary Figure 6 — Correlation of the AIM2 inflammasomes scores with the tumor immune dysfunction and exclusion scores. [file Image_6.tif]

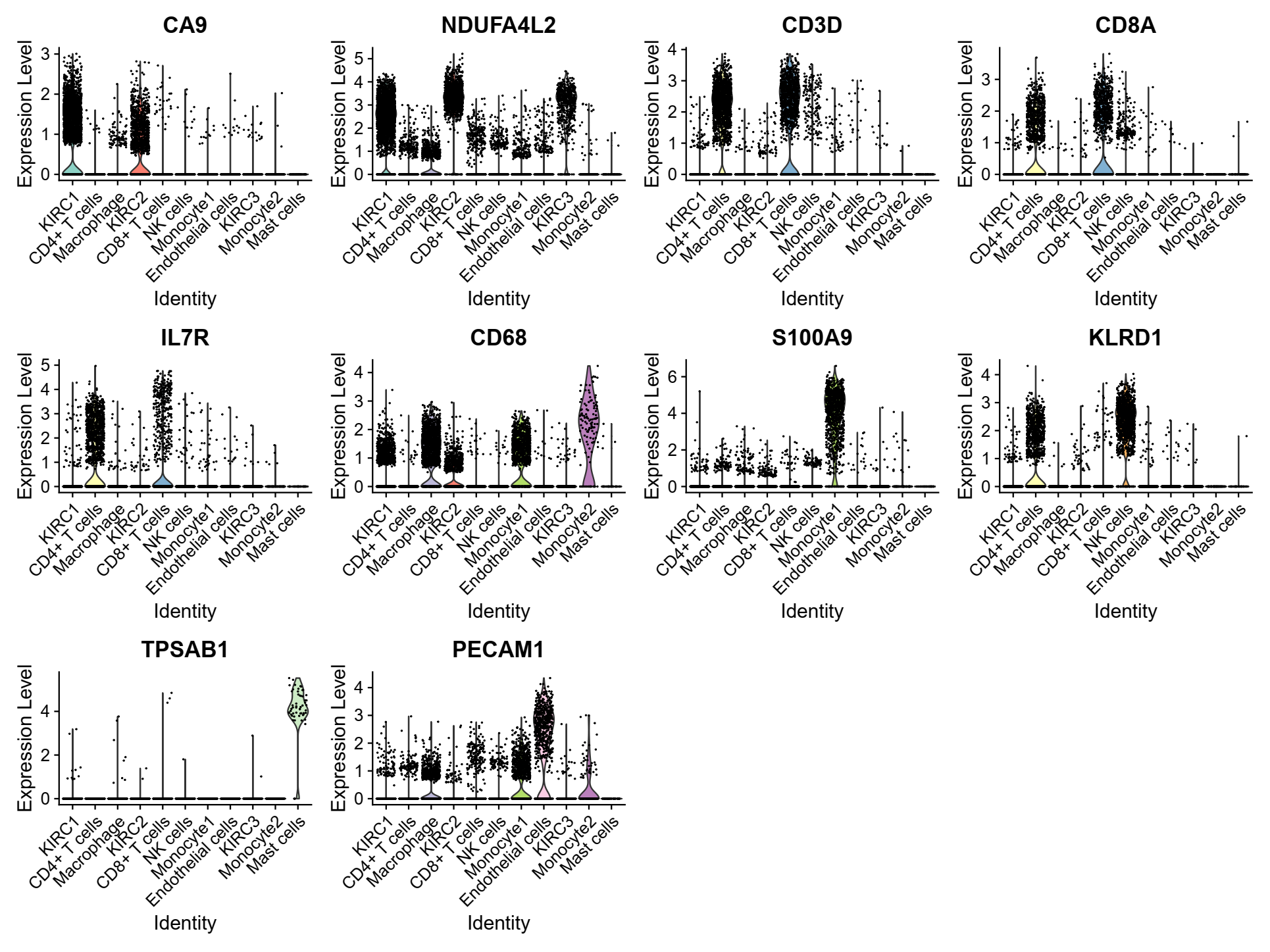

Supplement: Supplementary Figure 7 — Expression of the marker genes in different cells in the Kidney renal clear cell carcinoma tumor microenvironment. [file Image_7.tif]

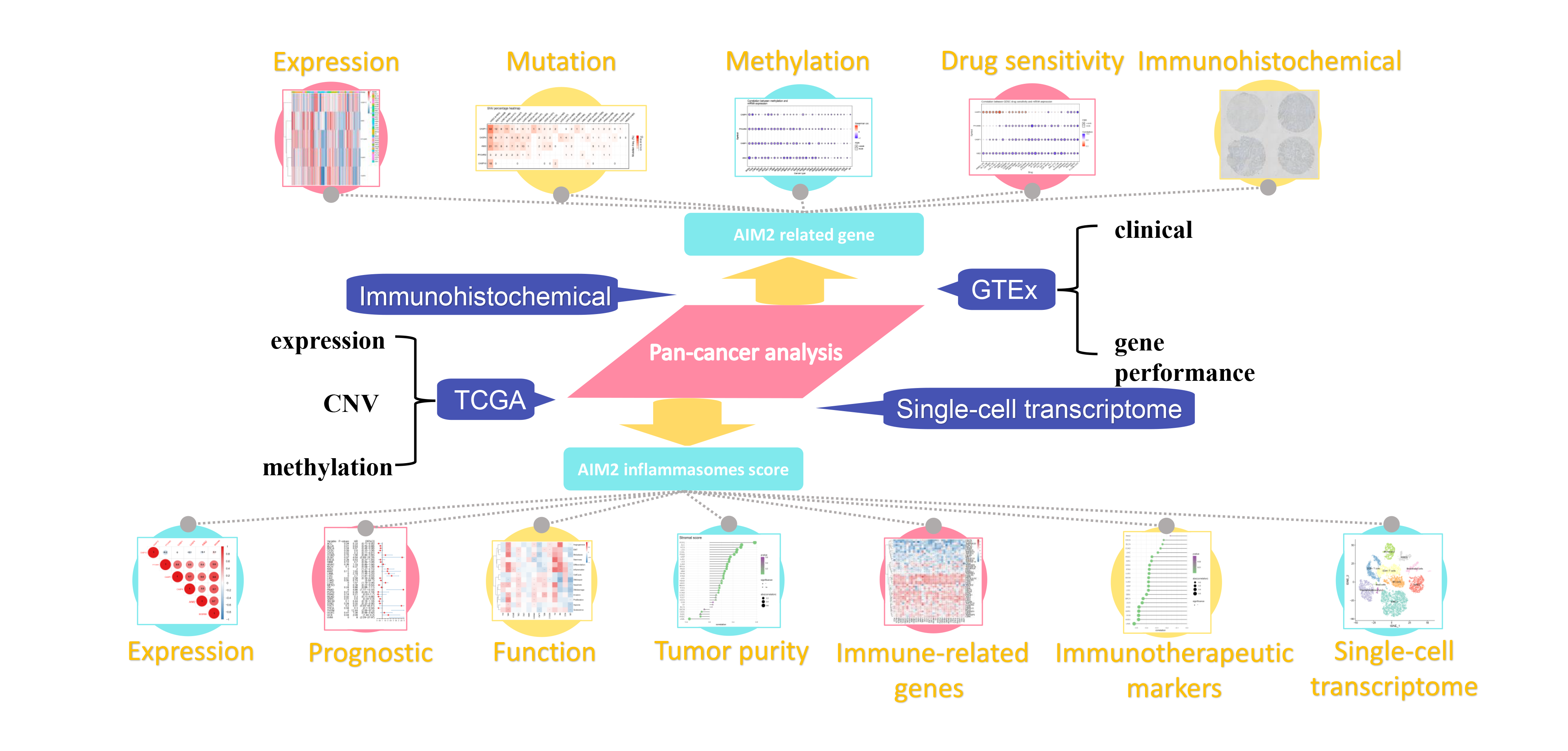

Supplement: Supplementary Figure 8 — The graphical abstract of the AIM2 inflammasomes. [file Image_8.tif]
